# Detailed Analysis of a Contiguous 22-Mb Region of the Maize Genome

**DOI:** 10.1371/journal.pgen.1000728

**Published:** 2009-11-20

**Authors:** Fusheng Wei, Joshua C. Stein, Chengzhi Liang, Jianwei Zhang, Robert S. Fulton, Regina S. Baucom, Emanuele De Paoli, Shiguo Zhou, Lixing Yang, Yujun Han, Shiran Pasternak, Apurva Narechania, Lifang Zhang, Cheng-Ting Yeh, Kai Ying, Dawn H. Nagel, Kristi Collura, David Kudrna, Jennifer Currie, Jinke Lin, HyeRan Kim, Angelina Angelova, Gabriel Scara, Marina Wissotski, Wolfgang Golser, Laura Courtney, Scott Kruchowski, Tina A. Graves, Susan M. Rock, Stephanie Adams, Lucinda A. Fulton, Catrina Fronick, William Courtney, Melissa Kramer, Lori Spiegel, Lydia Nascimento, Ananth Kalyanaraman, Cristian Chaparro, Jean-Marc Deragon, Phillip San Miguel, Ning Jiang, Susan R. Wessler, Pamela J. Green, Yeisoo Yu, David C. Schwartz, Blake C. Meyers, Jeffrey L. Bennetzen, Robert A. Martienssen, W. Richard McCombie, Srinivas Aluru, Sandra W. Clifton, Patrick S. Schnable, Doreen Ware, Richard K. Wilson, Rod A. Wing

**Affiliations:** 1 Arizona Genomics Institute, School of Plant Sciences and Department of Ecology and Evolutionary Biology, BIO5 Institute for Collaborative Research, University of Arizona, Tucson, Arizona, United States of America; 2 Cold Spring Harbor Laboratory, Cold Spring Harbor, New York, United States of America; 3 The Genome Center and Department of Genetics, Washington University School of Medicine, St. Louis, Missouri, United States of America; 4 Department of Genetics, University of Georgia, Athens, Georgia, United States of America; 5 Department of Plant and Soil Sciences and Delaware Biotechnology Institute, University of Delaware, Newark, Delaware, United States of America; 6 Laboratory for Molecular and Computational Genomics, Department of Chemistry, Laboratory of Genetics, University of Wisconsin Madison, Madison, Wisconsin, United States of America; 7 Department of Plant Biology, University of Georgia, Athens, Georgia, United States of America; 8 Department of Agronomy and Center for Plant Genomics, Iowa State University, Ames, Iowa, United States of America; 9 School of Electrical Engineering and Computer Science, Washington State University, Pullman, Washington, United States of America; 10 Université de Perpignan Via Domitia, CNRS UMR 5096, Perpignan, France; 11 Department of Horticulture and Landscape Architecture, Purdue University, West Lafayette, Indiana, United States of America; 12 Department of Horticulture, Michigan State University, East Lansing, Michigan, United States of America; 13 Department of Electrical and Computer Engineering, Iowa State University, Ames, Iowa, United States of America; The Salk Institute for Biological Studies, United States of America

## Abstract

Most of our understanding of plant genome structure and evolution has come from the careful annotation of small (e.g., 100 kb) sequenced genomic regions or from automated annotation of complete genome sequences. Here, we sequenced and carefully annotated a contiguous 22 Mb region of maize chromosome 4 using an improved pseudomolecule for annotation. The sequence segment was comprehensively ordered, oriented, and confirmed using the maize optical map. Nearly 84% of the sequence is composed of transposable elements (TEs) that are mostly nested within each other, of which most families are low-copy. We identified 544 gene models using multiple levels of evidence, as well as five miRNA genes. Gene fragments, many captured by TEs, are prevalent within this region. Elimination of gene redundancy from a tetraploid maize ancestor that originated a few million years ago is responsible in this region for most disruptions of synteny with sorghum and rice. Consistent with other sub-genomic analyses in maize, small RNA mapping showed that many small RNAs match TEs and that most TEs match small RNAs. These results, performed on ∼1% of the maize genome, demonstrate the feasibility of refining the B73 RefGen_v1 genome assembly by incorporating optical map, high-resolution genetic map, and comparative genomic data sets. Such improvements, along with those of gene and repeat annotation, will serve to promote future functional genomic and phylogenomic research in maize and other grasses.

## Author Summary

Maize is a major cereal crop and key experimental system for eukaryotic biology. Previous investigations of the maize genome at the sequence level have primarily focused on analyses of genome survey sequences and BAC contigs. Here we used a comprehensive set of resources to construct an ordered and oriented 22-Mb sequence from chromosome 4 that represents 1% of the maize genome. Genome annotation revealed the presence of 544 genes that are interspersed with transposable elements (TEs), which occupy 83.8% of the sequence. Fifty-one genes were involved in 14 tandem gene clusters and most appear to have arisen after lineage divergence. TEs, especially helitrons, were found to contain gene fragments and were widely distributed in gene-rich regions. Large inversions and unequal gene deletion between the two homoeologous maize regions were the main contributors to synteny disruption among maize, sorghum, and rice. We also show that small RNAs are primarily associated with TEs across the region. Comparison of this ordered and oriented sequence with the corresponding uncurated region in the whole genome sequence of maize resulted in improvements in TE annotation that will ultimately enhance detection sensitivity and characterization of TEs. Doing so is likely to improve the specificity of gene annotations.

## Introduction

The systematic genetic improvement of crop species achieved by plant breeders has been one of the great achievements of modern agriculture [1]. Agricultural systems face considerable challenges because inputs such as chemical fertilizers, pesticides, herbicides, water and arable land are becoming less available, affordable, or sustainable. In addition, because crops are adapted to relatively stable weather patterns, global climate change promises to disrupt crop production. Finally, agriculture now is being asked to provide not only food, feed, and fiber to a growing world population, but also to contribute substantially more to world fuel supplies. An enhanced understanding of basic crop biology is required to efficiently design and develop crops that can produce the higher sustainable yields with reduced inputs that are needed to satisfy current and future demands.

Maize has been, and continues to be, an important model system for basic biological research [2]. Because maize also is a crop, the resulting biological understanding is readily translated into crop improvement. In addition, knowledge gained from maize can be used to improve its relatives, including sorghum, sugarcane, and small grains.

The bulk of the maize genome is composed of highly repetitive transposable elements (TEs), that were first discovered in maize [3]. This initial TE identification was due partly to the ease with which associations with mutant phenotypes and high levels of TE activity could be made. Maize also was the first organism in which the quantitative contributions of TEs to genome structure were appreciated [4]–[8], leading to the current understanding that the major determinants of plant genome size are different rates of amplification and removal of TEs [9],[10]. TEs are largely responsible for the exceptionally high rates of rearrangement of both intergenic and genic DNA in plant genomes, observations first made in maize [11]–[14]. Thus, we now know that plant genome organization is primarily an outcome of the specificities and vagaries of TE action, and maize provides an excellent genetic platform for TE discovery and study.

Besides TEs, the maize genome is also unique in its recent polyploid origin. The cereals, such as maize, rice, sorghum, and wheat, shared a common ancestor some 50 million years ago (MYA) [15]. Their genomes are highly syntenic [16],[17] and the ancestor genome experienced an ancient whole genome duplication approximately 50–70 MYA [15],[18]. Although the maize genome is genetically and physically diploid with ten pairs of chromosomes, its genome contains a whole genome duplication resulting from the hybridization of two related maize progenitors [16],[19],[20]. Alignments of orthologous sorghum regions to two maize homoeologous regions indicated that sorghum and the two maize progenitors diverged at the same time about 11.9 MYA. The two maize progenitors may have hybridized as recently as 4.8 MYA [21].

The Maize Genome Sequencing Consortium (MGSC) was funded to provide draft quality sequence across the vast majority of the genome and finished high quality sequence (fewer than 1 error per 10,000 bases in genes and regulatory elements) in low-copy-number regions (e.g., genes and associated regulatory regions). To sequence the maize B73 genome, a minimum tiling path (MTP) of Bacterial Artificial Chromosome (BAC) and fosmid clones with known locations on the physical map [22] was sequenced to 4–6× fold coverage, assembled, sequence improved in low-copy regions, and annotated *via* automated approaches resulting in the first reference genome sequence for maize (i.e. B73 RefGen_v1) [23]. Over 32,000 genes were predicted, 99.8% of which could be placed on the integrated physical, genetic and optical maps. In addition, nearly 85% of the genome sequence was shown to consist of >1,000 transposable element families dispersed non-randomly across the genome.

Here, we report a detailed analysis of a contiguous ∼22-Mb region of the maize B73 genome, with sequence quality that has been further improved beyond the maize reference genome, by the incorporation of additional shotgun sequence and integration with the maize optical map, which serves as a pilot for the future whole genome analysis. The resultant analysis provides the most comprehensive study to date of a single region of the maize genome (∼1% of the 2300 Mb genome). These analyses demonstrate how additional automated and manual sequence improvement and annotation would affect the extraction of important biological information from the maize genome.

## Results/Discussion

### Genic, genetic, and physical features of a contiguous 22Mb sequence of maize chromosome 4

**Figure 1:**
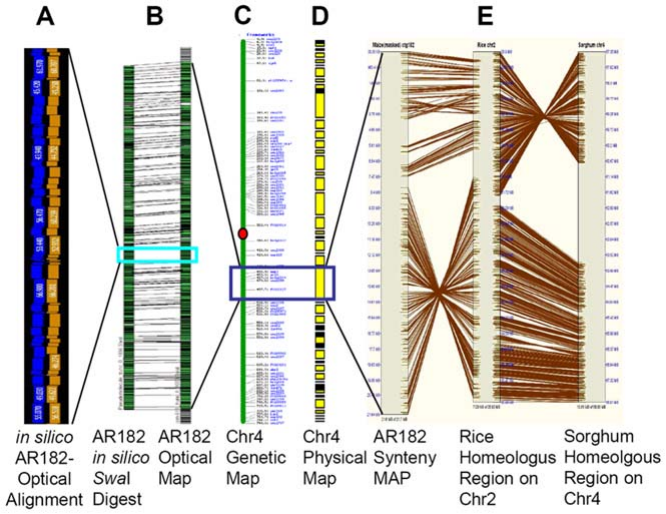
Physical and genetic features of AR182. Genetic (center), Optical (left), and physical and synteny maps (right) of AR182 are shown. (A) Magnified view of the AR182 pseudomolecule *in silico* restriction map (blue track) and Optical Map (gold track) alignment after finishing (each box = 1 restriction fragment); turquoise box demarcates zoomed region on (B) showing the entire alignment ∼22 Mb (∼1,000 ordered restriction fragments); (C) A simplified maize Chr4 genetic map; (D) The maize Chr4 physical map; (E) Overall syntenic relationships among maize, sorghum and rice with respect to AR182. This three-way synteny map was built by SyMap [83] from pseudomolecule to pseudomolecule comparisons.

Contig 182 in the B73 physical map of the maize genome [24] is located on chromosome 4 (Chr4), and was selected for analysis due to its large contiguous size (∼22 Mb) and exceptional colinearity with rice Chr2 (Figure 1C–1E).

Many interesting genes have been identified in this region (Table S1), such as r*f2b* (a paralog of a nuclear restorer of cytoplasmic male sterility that encodes an aldehyde dehydrogenase), *opaque endosperm 1* (*o1*), *dek31* (mutations in which result in defective kernels), *nitrite reductase 2* (*nii2*), *gl4* (a gene involved in the accumulation of cuticular waxes), and QTL related to ear length, diameter, grain yield, kernel length, weight, oil/protein/starch content, pest resistance and disease resistance [25]–[34]. Although several genes in this region have been cloned and functionally characterized, e.g., *nii2* [35], *rf2b* [36], and *gl4* [37] , none of the QTL have been functionally characterized.

Starting with the sequence-ready physical map [24], we selected a MTP of 176 BAC clones (Table S2) across contig 182 using the MTP analysis function of the Fingerprinted Contigs (FPC) program [38]. Standard shotgun sequencing protocols were employed for each BAC, and assembled sequences (∼4–6× redundancy) underwent K-mer analysis to identify repeats [39]. The remaining low-copy-number regions were finished to high quality. Pseudomolecules were constructed using BAC end sequences, overlap and scaffold information, and were adjusted and validated by alignment with the maize B73 optical map. (Figure 1A and 1B; [40]; see Materials and Methods section for details.) The final sequence contained 21,702,972 bp in 907 uninterrupted sequence blocks, herein referred to as accelerated region 182 (AR182). The contig N50 is 57,261 bp, and the scaffold N50 is 160,621 bp.

In this region, there are 178 genetic markers (Table S3) from bin 4.06 to bin 4.08 in the IBM2 2008 Neighbors maize genetic map (http://www.maizegdb.org/map.php)—a consensus map compiled from all available maize mapping populations. Among the 150 markers with sequence information, 124 were identified in AR182, and 18 were located in flanking contig 181 (19 markers) or neighboring contig 197 (1 marker). Of the remaining eight markers, all were placed in other regions of the maize genome. Seven of these eight markers are multiple copy RFLP markers and could not be detected on maize Chr4 at e^−5^, perhaps because these restriction fragment length polymorphism (RFLP) markers were incorrectly mapped, or are not present in the B73 genome. Two companion studies [41],[42] used resequencing and comparative genome hybridization to demonstrate that maize exhibits high frequencies of haplotype-specific sequences (Presence/Absence Variation). Many of these PAVs may have arisen via a consequence of the movement of transposable elements carrying genes or gene fragments. This finding, in combination with our use of a consensus map derived from multiple mapping populations may explain the absence of the eight genetic markers in AR182. Among the 13 framework markers with solid genetic positions, 12 had corresponding sequences in AR182. With the exception of two adjoining markers (*umc104a* and *mmp147*) with switched positions, all other markers had the same order in the physical map as in the genetic map. The ratio of genetic to physical distance across AR182 averaged 4.4 cM/Mb (Table S4), somewhat lower than the previously-estimated genome average of 5.5 cm/Mb [24].

### Transposable elements and their contributions to maize genome evolution

Transposable elements (TEs) are the most numerous and unstable components of the maize genome, and of all other complex plant genomes studied to date. In addition, TEs significantly complicate genome assembly and annotation because they are often repetitive, can be located in and around genes, and often encode ORFs that are easily mistaken for standard plant genes [43]. Because many of these TEs, especially the long terminal repeat (LTR) retrotransposons, are large and very similar in sequence due to their recent amplification, repetitive TEs are a major source of gaps and misassembled contigs in complex plant genomes. The simplest way to minimize the negative impact of TEs on gene discovery and annotation is to initially describe all of the TEs in a region. This allows TEs to be computationally masked, thereby providing a residual sequence that can be carefully analyzed. Structure-based searches are especially useful for the discovery of novel TEs, especially given that many are both low in copy number and represented in EST libraries.

TE and other repeats were sought within the assembled sequence of AR182 by several independent approaches. Repeats *per se* were identified using an oligonucleotide counter that searched for the representation of all possible 20-mers in 1,124,441 whole genome shotgun reads (1,088,525,270 nucleotides; ∼0.45 genome equivalents [39]). Repeats also were found by homology to known repeats in the MIPS REdata database (v4.3) [44] and TE exemplar databases [45]. Finally, structure-based searches were employed to identify novel TEs, including those that exist in low copy numbers. These structure-based search processes rely on the unique characteristics of particular classes of TEs, especially their end structures, but require significant manual curation to confirm the validity of any candidate TEs that are identified.

The most abundant repeats identified were the LTR retrotransposons, which were found to constitute about 74.6% of the assembled sequence. The identified LTR retrotransposons were divided into 237 families. Intact elements (i.e. with 2 LTRs and the appropriate internal sequences) were found in this region for 47 of these families. One hundred and eighty-one of these families were represented in maize EST libraries (data not shown). The specific elements present, their copy numbers and their relative coverage on AR182 are provided in Table S5. As seen in earlier studies of maize [4] and other large plant genomes [46]–[49], most of these elements are inserted into each other in nested arrangements with the oldest elements at the base of the stacks (e.g. Figure S1A and Figure S2A). Two other classes of retroelements, LINEs and SINEs, were located in this region, providing 1.1% and 0.03% of the assembled sequence, respectively (Table S6).

In AR182, *Copia*-like retrotransposons were found to be over-represented (29.2%) relative to the entire maize genome (23.7%), while *Gypsy*-like retrotransposons were found to be under-represented (38.9 vs 46.4%; Table 1). These results agree with earlier studies [50],[51] showing that different LTR retrotransposons preferentially accumulate in different areas of the maize genome. Although all of these high-copy-number LTR retrotransposons appear to prefer to insert into each other rather than into genes, they also distinguish LTR retrotransposon clusters that are near genes and those that are in largely gene-free regions like pericentromeric heterochromatin. In yeast, this class of elements finds insertion sites by association between the element-encoded integrase and specific heterochromatin proteins [52]. The presence of chromodomains in some, but not all, plant LTR retrotransposons [53] suggests a similar targeting mechanism.

**Table 1:** Summary of transposable elements^a^.

		No. of TE families			No. of TEs (×1000)			Coverage(Mb)			Fraction of genome		
Class	Superfamilies	B73	AR182	AGP182	B73	AR182	AGP182	B73	AR182	AGP182	B73	AR182	AGP182
**Class I**	**LTR/Copia**	109	77	74	404	4.572	4.734	484	6.333	6.421	23.7	29.2	28.8
	**LTR/Gypsy**	134	69	69	477	3.488	3.691	948	8.448	8.668	46.4	38.9	38.9
	**LTR/Unknown**	163	91	89	222	2.294	2.361	92.9	1.19	1.274	4.5	5.5	5.7
	**LINE**	31	31	31	35	0.31	0.31	20	0.225	0.224	1	1	1
	**SINE**	4	2	2	2	0.028	0.024	0.5	0.007	0.007	0	0	0
	**Subtotal**	441	270	265	1,140	10.69	11.12	1,546	16.2	16.594	75.6	74.6	74.4
**Class II**	**CACTA**	156	66	65	12.4	0.092	0.095	64.7	0.576	0.586	3.2	2.7	2.7
	**hAT**	230	178	181	31.8	0.42	0.432	23.4	0.31	0.317	1.1	1.4	1.5
	**Mule**	155	88	88	12.9	0.163	0.166	20.2	0.219	0.221	1	1	1
	**MLE/Stowaway**	127	45	47	14	0.165	0.166	2.3	0.025	0.025	0.1	0.1	0.1
	**PIF/Tourist**	179	137	137	49.7	0.579	0.588	19.8	0.229	0.232	1	1	1.1
	**Helitron**	8	6	6	22	1.149	1.299	45.5	0.653	0.647	2.2	3	3
	**Subtotal**	855	520	524	143	2.568	2.746	176	2.012	2.028	8.6	9.2	9.4
**Total TEs**		1,296	790	789	1,283	13.26	13.866	1,722	18.22	18.622	84.5	83.8	83.8

**a** data of genome and AGP182, the AR182 corresponding sequence in B73 RefGen_v1 are from Schnable et al. **[23]**.

DNA transposons also were well represented in this region (Table 1), including 92 CACTA elements (66 families), 420 *hAT* elements (178 families), 744 MITEs (182 families), 163 MULEs (88 families) and 1,149 mostly fragmented *Helitrons* (6 families), and each class comprised between 1–3% of AR182. Few of these elements are likely to be autonomous (encoding all the functions needed for transposition). For seven of the CACTA families, we found at least one copy with intact open reading frames. Four *Helitrons* were found to contain apparently full-length *Rep/helicase* genes with protein products believed to be necessary for transposition.

Unlike the highly abundant LTR retrotransposons, the MITEs, *Helitrons*, CACTAs and MULEs primarily were found to be associated with genes (Figure S3). This is also the case for small SINE retroelements, as most copies present in the AR182 region were found in gene introns. The preferential insertion and/or retention of these lower-copy-number elements in these presumably euchromatic regions has the advantage of maintaining their potential for expression. However, by locating in recombinationally active regions near genes [54],[55] their potential to contribute to genome rearrangements is increased.

Perhaps the most amazing characteristic of the maize genome is the incredible number of gene fragments that are found inside TEs. Several classes of TEs have been found to acquire and transpose fragments of normal cellular genes, with MULEs and *Helitrons* particularly active in this regard [14],[56]. AR182 was found to contain 20 LTR retrotransposons with apparent gene fragment insertions, plus 9 MULEs, 5 CACTA TEs, and 187 *Helitrons* with one or more acquired gene fragments (Table S7). The capture of gene fragments by LTR retrotransposons and CACTA elements has been reported before [57]–[60], but the extent has not been known for any plant genome. The analysis of AR182 demonstrated that this is a common phenomenon in maize.

In purely automated genome annotations, most or all of these fragments would have been counted as genes. Hence, in this region, 1,009 rather than 544 genes would have initially been predicted, and extrapolations to the entire maize genome nearly would have doubled overall gene content. Combining this error with the common error of annotating TE-encoded transposition genes as standard plant genes principally is responsible for the two-fold or more errors in gene content that have sometimes occurred in plant genome analysis [43]. Beyond the complications they create for gene discovery and annotation, the gene fragments within TEs also generate many questions about their possible contributions to host cell biology. Although the rapid rate of removal of unselected DNA from plant nuclear genomes [9] suggests that the great majority of the gene fragments and multi-gene chimeras within TEs rapidly become extinct, even the rare creation of a novel gene by the process of exon shuffling [61] could have enormous biological significance. Many cases of “transposon domestication” [62], where all or part of a TE has been co-opted by the host organism to perform an important biological function now have been reported. The acquisition of gene fragments from multiple loci, and their fusion with each other and with standard TE proteins, should only increase the potential for valuable novelty and domestication. Equally important, the epigenetic silencing of TEs by siRNAs [63],[64] predicts that many of the gene fragments inside TEs could contribute to the pool of siRNAs, and thereby acquire regulatory roles over the genes from which they were derived. Perhaps this is the mechanism of origin of some microRNAs, as fragments created by TEs that have evolved to encode specific small RNAs that regulate the source gene [65].

**Figure 2:**
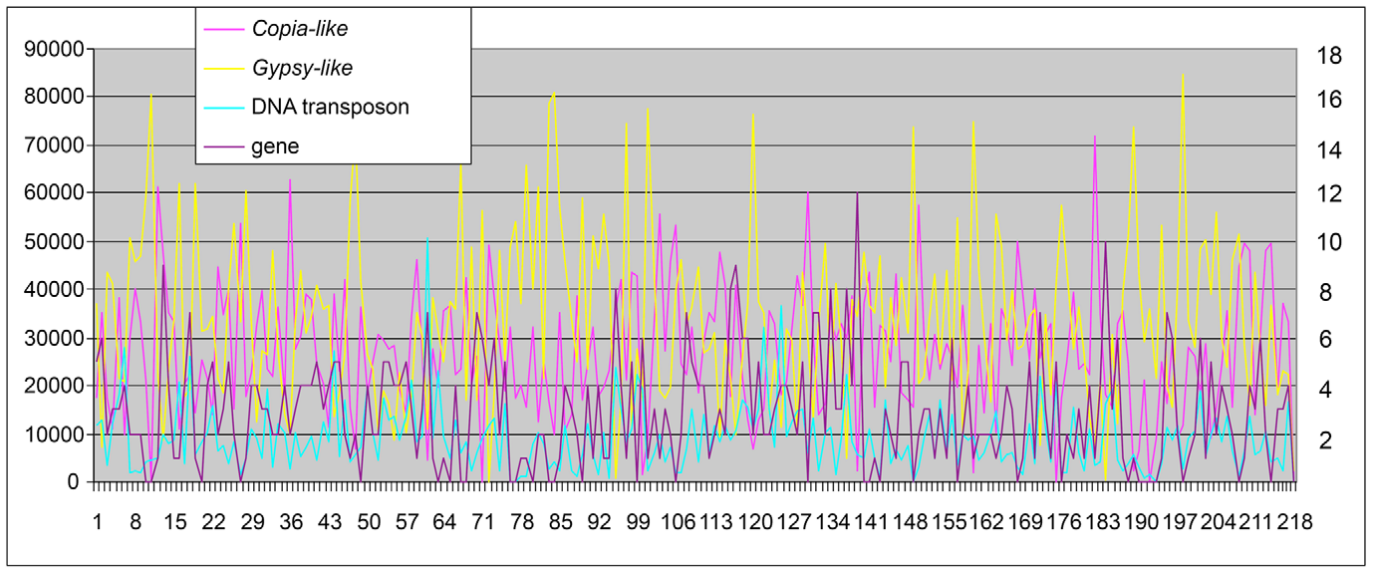
TE and gene distribution along AR182. The distribution was constructed based on nucleotide length of the related TE in 100-kb sliding windows. The numbers at the left vertical axis represent the nucleotide length of related TE classifications. The numbers in the right axis are the gene number counts.

The distributions of these TEs across the region appeared uneven when viewed at the level of the entire AR182 (Figure 2). Among LTR retrotransposons, the concentrations of *Gypsy*- and *Copia*-like elements were correlated inversely. On a smaller scale, specific TE arrangements were found to be highly non-random. LTR retrotransposons primarily were inserted into each other and away from most genes, while DNA transposons, such as CACTAs, *Helitrons*, and MULEs, or small retroelements such as SINEs, were near genes. It should be noted that novel TEs that are low in copy number or have no intact copies here or elsewhere in the maize genome will still have been missed in this annotation process, so it is expected that this will cause some under-estimate of TE number and an over-estimate of gene number.

### Gene identification and characterization

Annotation of protein-coding genes was based predominantly on extrinsic evidence, using a gene building process adapted from Ensembl [66],[67]. Sources of evidence included sequences from maize full-length cDNAs [68] (http://www.maizecdna.org/) as well as ESTs and proteins. *Ab initio* predictions were included only where they did not overlap with evidence-based genes, or where overlap allowed extension of coding sequences. Although known repeats were masked prior to annotation, additional measures (see Materials and Methods) were needed to screen TEs, a common source of false positive predictions in plants [43]. Manual methods also were used to identify and remedy falsely split or fused gene models, though these were relatively rare. The resulting gene set includes 544 annotated loci, of which 514 were evidence-based, including 160 by full-length cDNAs (Table S8). Overall, AR182 has a gene density of 25 genes per Mb. Gene content in the 2045 Mb RefGen_v1 whole genome assembly was estimated at between ∼37,000 and ∼39,000, giving a gene density of 18 to 19 genes/Mb [23]. Hence, AR182 is relatively gene-rich compared to the genome overall. Seven pairs of genes were found to be overlapping, and this conclusion is supported by full-length cDNA or protein homologs in other species. In rice, the presence of overlapping genes is relatively common and most are caused by transcripts using the promoter or enhancer of LTRs in a retrotransposon (Wei and Wing, unpublished). Given the large number of LTR retrotransposons in maize, it would not be surprising if the observation of overlapping genes is be common in maize. Among the non-overlapping genes, the intergenic spaces in 246 (45.3%) of the 543 gene spaces were less than 10 kb while 240 (44.2%) genes were separated by more than 20 kb. Fifty-four of the intergenic regions were greater than 100 kb, with the largest being 530 kb (Figure S4). Most of these large intergenic regions are filled with nested LTR retrotransposons (Figure S1 and Figure S2).

Gene, exon, and intron lengths, as well as number of exons per gene, were found to be within previously estimated ranges [69],[70], as shown in Table 2. To make comparisons with other cereals, we selected 341 ortholog sets having three-way colinearity within syntenic regions of maize, sorghum and rice (Table 2). Exon lengths were relatively invariable across species, consistent with previous findings [70],[71]. This contrasts with introns, which averaged 229 bp, 361 bp, and 498 bp for rice, sorghum, and maize, respectively. Haberer et al. [70] had previously reported this trend and also found examples of introns harboring TEs, suggesting that such insertions were responsible for inflated intron sizes in maize, which is consistent with earlier reports of TE and retrotransposon insertions within maize introns [72],[73]. To further examine this hypothesis, we directly compared orthologous introns among maize, sorghum, and rice. Introns were paired based on their conserved position between flanking mapped exons (see Materials and Methods). When introns of less than 1 kb were considered, lengths between pairs were strongly correlated (Figure S5). The correlation was greater between maize-sorghum than between maize-rice, consistent with their more recent divergence. However, maize had more large introns, leading to discrepancies in paired intron lengths. For example, 2.6% of maize introns were observed to be larger than 3 kb, whereas this number was only 0.47% in sorghum and 0.17% in rice (Figure S6). Length discrepancies in which the maize intron exceeded the length of its cross-species partner by more than 1 kb occurred in 4.7% of mapped intron pairs, whereas the reverse was true in only 0.55% of cases (Figure S7). Figure S8 shows a clear linear relationship between length discrepancies in positionally conserved introns and repeat content within such maize introns. All told, about 2.4% of maize introns harbor repetitive sequences exceeding 1 kb or greater (an example of nested LTR retrotransposons in an intron shown in Figure S9) and 11% of intron-containing maize genes have at least one intron with this characteristic. That these genes are active is strongly indicated by evidence derived from GenBank mRNAs/full-length cDNAs.

**Table 2:** Comparison of maize, sorghum, and rice genes^a^.

Parameter	Gene set^b^	Mean	Std dev.	Median	Max
**Gene length (kb)**	Zm (all)	3.5	3.7	2.4	29.2
	Zm (syn)	3.4	3.8	2.1	23
	Sb (syn)	3.1	2.5	2.4	14.5
	Os (syn)	2.9	2.2	2.3	12
**Exon length (bp)**	Zm (all)	306	394	156	3,389
	Zm (syn)	251	337	134	3,087
	Sb (syn)	246	329	133	3,090
	Os (syn)	243	330	131	3,627
**Intron length (bp)**	Zm (all)	482	1079	151	18,487
	Zm (syn)	498	1123	144	18,487
	Sb (syn)	361	532	149	8,794
	Os (syn)	329	478	147	9,436
**Exon count**	Zm (all)	5	4.6	3	37
	Zm (syn)	5.3	4.9	4	28
	Sb (syn)	5.6	5.3	4	28
	Os (syn)	5.6	5.2	4	28

**a** Where multiple transcripts are described for a gene, the one with the longest coding sequence was used.

**b** Zm = maize; Sb = sorghum; Os = rice. “all” refers to the entire set of 544 maize genes; “syn” refers to a set of 341 presumed orthologous and syntenic genes in each species. For consistency, only exons and introns within the CDS were characterized in the “syn” set.

Besides these protein-coding genes, five miRNA genes in four families were computationally identified. The overall density of miRNAs in this region is 3 fold higher than the average genome distribution and all 5 genes have evidence of expression based on small RNA libraries [74].

### Synteny analysis across maize, rice, and sorghum in AR182

**Figure 3:**
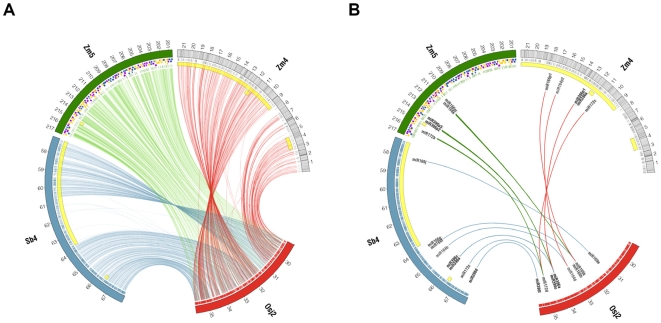
Comparative mapping of protein-coding and miRNA genes in orthologous segments of the rice, sorghum, and maize genomes. Abbreviations: Osj2 = rice (japonica) chromosome 2; Sb4 = sorghum chromosome 4; Zm4 = maize chromosome 4 (AR182); Zm5 = maize chromosome 5 (homeologous region). All mappings are drawn relative to rice as a common reference. Genes are shown as tick marks in the outer radius of correspondence lines. Inversions are indicated with yellow highlighting. For Zm4 the density of repetitive sequence is shown in gray. Zm5 mappings are to individual BACs (boxes) projected onto the FPC map. (A) Mappings of protein-coding genes based on reciprocal best hit. (B) Mappings of miRNA genes based on family membership.

Previous studies have shown that extensive genetic colinearity and synteny exist among the maize, rice and sorghum genomes [16], [24], [75]–[81]. All those studies were based on either genetic markers or short contiguous sequence analysis. In this study, four sequence-to-sequence comparisons were performed among the three species, including maize-rice, maize-sorghum, rice-sorghum, and maize-maize analysis using BLASTZ [82] and the Synteny Mapping and Analysis Program (SyMAP, [83]). AR182 on maize Chr4 was found to align with rice Chr2 (29,020,340–35,806,283; Figure 1E) and sorghum Chr4 (57,193,840–60,617,265 and 63,725,383–67,939,454; Figure 1E), and maize Chr5 from part of ctg250 to ctg254 ([24]; Figure S10; clone list in Table S9). While Figure 1E shows a pairwise pseudomolecule-to-pseudomolecule comparison of sequences, Figure 3 shows a comparative map based on homologous genes within these regions. The map in Figure 3 uses rice as a common reference because rice has been consistently identified as containing a relatively stable genome that closely resembles the ancestral state [78]. In the syntenic regions, there were annotations of 544 maize genes, 825 rice genes, and 847 sorghum genes. A higher level of synteny was observed between rice and sorghum than between maize and rice. Indeed, 686 (83.2%) of the 825 rice genes in the corresponding region were found to be syntenic to sorghum, while 375 (45.5%) of the rice genes were syntenic to the maize region. The same was true in that 685 (80.9%) of the 847 sorghum genes were syntenic to rice, while 362 (66.5%) of the 544 maize genes were syntenic to rice. Direct comparisons between maize and sorghum in AR182 revealed that 394 (72.4%) maize genes were syntenic to sorghum, while 396 (46.8%) sorghum genes were syntenic maize genes (Figure S11). Of course, any false positive gene annotations of TEs as genes in any of these regions [43] would be perceived as having non-syntenic relationships. It should be noted that the selected AR182 region is highly collinear with rice, however, at the whole genome level, maize is probably less syntenic with rice than estimated here. All five of the miRNA genes were found to be syntenic (Figure 3B) to a corresponding region in rice and sorghum. Four of the genes also were retained on the homeologous arm.

Two hundred and forty-one genes maize genes (44.3%) from AR182 were syntenic to its homeologous region on Chr5. This result is quite different from a previous study that showed only 20–28% of the genes located on duplicated and sequenced regions of Chr1S and 9L [69] were syntenic. At the genome level, 25% of the conserved maize genes maintained their homeologous copy [23]. These results suggest that the degree of genome “fractionation” (i.e., loss of one homeologous copy from the ancestral *Zea* tetraploid formed 5–12 MYA [21]) can be very different in various regions of the genome. As expected, 337 (75%) of the 450 rice genes that are not syntenic to AR182 were observed to be syntenic and colinear in the corresponding maize Chr5 region. In total, 726 (88%) of the 825 rice genes are syntenic to at least one of the two maize syntenic regions. These data strongly support previous proposals that deletion of redundant homologous maize genes is the major factor that disrupts colinearity between maize and other species [81],[84].

### Genome rearrangement and tandem duplication among maize, rice, and sorghum

Comparisons between maize-maize, maize-rice, maize-sorghum, and rice-sorghum revealed several rearrangements. Regions syntenic to AR182 from both maize and sorghum contain large inversion breakpoints that formed independently after the maize-sorghum lineage split (Figure 1E). By contrast, colinearity between the maize homeologous region in Chr5 and the rice genome spans the entire region, with no apparent rearrangement (Figure S10), indicating that the inversion on maize Chr4 occurred after the ancestral *Zea* tetraploidization. Inversions in both maize and sorghum extend beyond the region under study. For sorghum, the inversion breakpoints occur at ∼57.1 and ∼63.7 Mb. Because the first breakpoint lies outside AR182, the inversion introduces an ∼3.1 Mb flanking sequence, bearing some 375 genes, for which homologous genes are absent from the other genomes within the scope of the region. For maize Chr4, the first inversion breakpoint is at ∼8.5 Mb, while the second occurs downstream within ctg184 (not shown). This left a gap in rice within which ∼68 genes map to ctg184 rather than AR182. Additional, possibly overlapping, inversions occur within maize Chr4, ∼2.9 to 4.4 Mb, and this also arose after the whole genome allotetraploidization. Finally, a smaller inversion is conserved in both sorghum and the two homologous regions of maize, corresponding to coordinates ∼34.6 to 34.7 Mb in rice. This rearrangement occurred after the rice-sorghum/maize lineage split but its lineage of origin is unclear.

By using rice as a reference genome, one can infer the timing of each rearrangement. All of the rearrangements were observed to be specific to each genome and none were shared among the genomes. Previous studies showed that rice diverged from maize and sorghum about 50–70 million years ago, the ancestors of maize and sorghum diverged about 12 MYA, and the two ancestors of current maize hybridized about 4.8 MYA [15],[21]. Combining the evolutionary data of the species with comparisons in AR182-rice-sorghum and maize AR182-rice-maize Chr5, one can infer that these inversions occurred after lineage divergence. The maize Chr5 region demonstrates perfect synteny to rice and therefore preserves the original order and orientation of the ancestors of maize and sorghum. The sorghum genome experienced the inversion after divergence with the ancestors of maize, while the two larger inversions in AR182 of maize Chr4 perhaps arose during genome shuffling after the tetraploid progenitor of maize originated. In sequence divergence (Ks) analysis (see below), indistinguishable distances were observed between sorghum/maize and maize/maize homeologes, indicating a very similar date of lineage divergence with ancestral maize duplication; consistent with the ∼12 MYA timing predicted in a previous study [21].

Extensive tandem gene duplication has been found in Arabidopsis (17%; [85]) and rice (14–29%; [86]). In AR182, 51 (8.1% of the total) genes were found to be involved in 14 tandem duplication clusters with 2–19 genes in each cluster. Most (9) of the clusters have only two genes. The largest gene family in the region is the 19-member DUF1754 superfamily. This gene family is present in most eukaryotic genomes, including those in mammals, birds, fish, insects, fungi and plants. The biological function of the DUF1754 superfamily is unknown. There is one gene copy in most species (such as human, chimpanzee, chicken, rice and Arabidopsis), two copies in several others (mouse, sorghum, and popular), and seven copies in the bovine genome. The gene was not detected in nematodes. The 19 members in AR182 are distributed in a 1.16 Mb region and are interrupted by twelve other genes. Additionally, there are two other family members in maize, located on Chr3 and 8.

Interestingly, 8 of the 14 gene clusters are not syntenic with either rice or sorghum. In the corresponding co-linear rice region, there are 105 genes (10.6%) involved in 33 duplication clusters with each cluster varying from 2 to 8 genes. Nineteen of the 33 clusters involved only 2 genes and 20 of the 33 clusters have no syntenic relationships to maize AR182. Ninety-two (10.0%) of the sorghum genes were observed to be involved in 37 tandem duplication clusters, with 2 to 7 genes in each cluster. Twenty-six of the 37 sorghum gene clusters have 2 genes and 12 of the 37 clusters have no syntenic relationships with maize.

The synteny data for tandem gene duplication in rice, sorghum, and maize indicate that most of the tandem duplication occurred after lineage divergence, in agreement with previous studies in *Drosophila* that tandem duplicated genes tend to be younger with lower survivorships [87].

### High frequencies of mutation and truncation among non-syntenic genes

**Figure 4:**
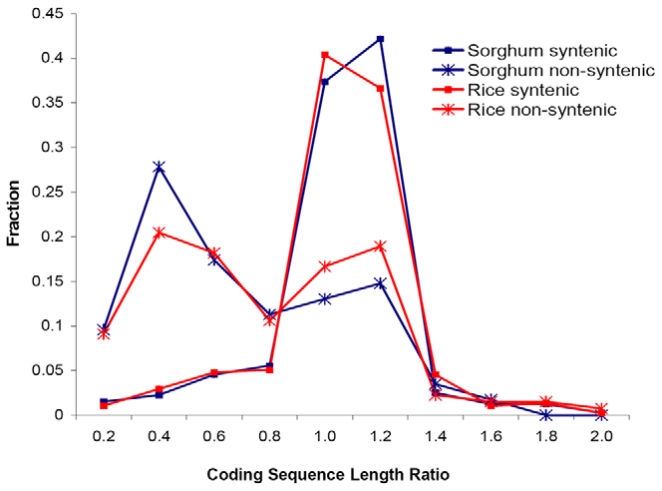
Distribution of truncated genes among syntenic and non-syntenic maize loci. Protein coding length ratio (length of maize/length of sorghum or rice) between highest scoring homologs is used as a measure of truncation. Non-syntenic loci contrast with syntenic loci in showing a bimodal distribution.

We are aware of at least two possible processes that would result in non-synteny: gene mobilization from one location to a new location and corresponding gene loss in the other species. Because most genes that are non-syntenic relative to rice are also non-syntenic relative to sorghum, the more parsimonious explanation is that these non-syntenic maize genes were mobilized from elsewhere in the genome. As shown above, mobilization of genes, particularly by transposons such as *Helitrons* and Pack-MULEs, frequently result in fragmentation of the amplified/transposed copy [14],[88]. To examine this phenomenon, we calculated the ratio of the CDS (coding sequence) length of the maize gene to that of its best scoring rice or sorghum homolog (ortholog for syntenic relationships). While syntenic genes have a single CDS ratio peak centered at one, non-syntenic loci have a bimodal distribution, with a second peak centered at 0.4, indicative of frequent truncation (Figure 4). As relates to sorghum, 68% of non-syntenic maize genes have a CDS ratio of less than 0.8 whereas only 14% of syntenic loci do. Thus, a substantial proportion of non-syntenic genes are fragmented, consistent with a mechanism of gene mobilization and the likelihood that these are truncated pseudogenes [51].

**Figure 5:**
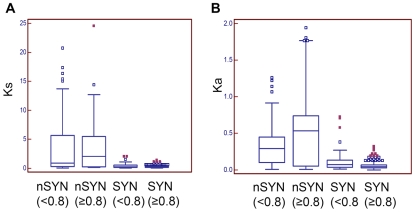
Box-plots showing divergence rates among syntenic (SYN) and non-syntenic (nSYN) maize genes relative to their best scoring homolog in sorghum. (A) Ks. (B) Ka. Genes were categorized by CDS length ratio using a threshold of 0.8 (maize CDS length / sorghum CDS length). Sample sizes: nSYN(<0.8) n = 68; nSYN(≥0.8) n = 32; SYN(<0.8) n = 54; SYN(≥0.8) n = 340.

To further characterize these, synonymous (Ks) and non-synonymous (ka) mutation rates (Ks) were measured relative to their best-scoring homologs in sorghum. For this analysis, six potential false-negative syntenic genes were identified by TBLASTN alignment to sorghum, possibly missed due to omission of these genes in the sorghum annotation. Figure 5 shows distributions of Ks and Ka, for maize loci, stratified by synteny relationship and by evidence of truncation using a CDS length ratio threshold of 0.8. Large differences were seen between syntenic genes and non-syntenic genes for characteristics of both Ka and Ks. The Mann-Whitney test [89] for non-parametric data showed that these differences are significant. The median Ks is 0.2352 (95%CI 0.2131 to 0.2674) for syntenic loci compared to 0.9769 (95% CI 0.7153–1.5543) for non-syntenic loci (P<0.0001). Ks was significantly different even when considering only genes with a CDS length ratio ≥0.8, For this class the median Ks for syntenic loci was 0.2326 (95%CI 0.2130 to 0.2681), compared to 2.0389 (95% CI 0.3114 to 3.7455) for non-syntenic genes (P<0.0001). Thus, truncation itself is not associated with elevated Ks values. Indeed, the Ks for non-syntenic loci having a CDS length ratio <0.8 (median = 0.9064 (95% CI 0.5168 to 1.2777) is not significantly different from those having CDS length ratio ≥0.8 (P = 0.6310). Because Ks approximates mutation rate [90],[91], this result suggests that non-syntenic mappings have a more ancient relationship than do the orthologous relationships found in syntenic genes. The rate of non-synonymous mutation (Ka) likewise is elevated among non-syntenic genes. The median Ka for syntenic loci is 0.0442 (95%CI 0.0411 to 0.04889) compared to 0.2965 (95%CI 0.2426 to 0.3981) for non-syntenic loci (P<0.0001). It is clear that non-syntenic loci have vastly different properties compared to syntenic genes and that the identified sorghum homologs of non-syntenic maize genes cannot be regarded as orthologs.

### Small RNA analysis

To determine the extent to which the sequence of AR182 may contribute to, or interact with, the small RNA population expressed by the whole maize genome, five small RNA libraries representing different maize tissues and genetic backgrounds were analyzed (see Materials and Methods for details). Three libraries (B73-zma1, B73-zma2 and B73-zma3) were constructed using small RNA fractions from young leaves, immature ears and immature tassels, respectively, of a B73 genotype. The remaining two libraries (K55-wt and K55-mop1) were previously described by Nobuta et al. [92] and include small RNAs from immature ears of wild-type and *mop1-1* maize, respectively, in a K55 background. The *mop1* gene was shown to encode an ortholog of *Arabidopsis* RNA-DEPENDENT RNA POLYMERASE 2 (RDR2) and is required for the establishment of paramutation and the maintenance of transcriptional silencing of transposons and transgenes [93],[94].

On average, the proportion of distinct small RNAs matching the sequence of AR182 at least once was 12% per library, corresponding to a range of ∼147,000 to ∼380,000 different small RNA sequences (Table S10). The leaf tissue libraries exhibited the lowest complexity, with approximately half the rate of matched, distinct sequences compared to any other sample (which all represented reproductive organs) and a distinct to total reads ratio of 16% in this library (compared to more than 30% in the other libraries).

All of the libraries exhibited a similar pattern of size distribution with two prominent peaks at 22 nt and 24 nt respectively (Figure S12); as expected, in this contig, small RNAs in K55-mop1 presented a strikingly lower proportion of repeat-associated 24-mers compared to K55-wt. Moreover, consistent with prior reports, the 22-nt class predominantly associated with high-copy repeats was more abundant than the 21-mers, both when distinct numbers and total abundances were taken into account [92]. Based on these observations, the population of small RNAs matched to AR182 demonstrated small RNA match rates and patterns consistent with other analyses of sub-genomic portions of the maize genome [92].

**Figure 6:**
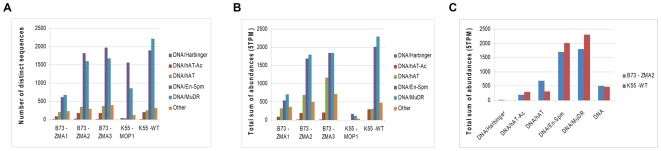
Distributions of DNA transposons and their related small RNAs. (A) Number of distinct DNA transposon-related small RNAs under different genetic backgrounds. (B) The total number of DNA transposon-related small RNAs under different genetic backgrounds. (C) Small RNAs discrepancies between B73 and K55-wt backgrounds.

Among the small RNAs matching to AR182, 54% had more than two hits in a set of 60 Mb of maize contigs (including this contig from Chr 4, plus two other contigs from chr 1, and 9), suggesting that most small RNAs may be derived from repetitive elements. First, 25–38% of the unique signatures from each library were found to match tandem repeats (Table 3), which are known substrates for small RNA biosynthesis [95]. Next, to investigate in detail the fraction of small RNA originating from transposons, five principal families of DNA transposons were examined. These families included *Harbinger*, *hAT*, *En-Spm*, *MuDR*, two superfamilies of LTR retrotransposons (*Copia* and *Gypsy*), and a family of non-LTR retrotransposons (LINE1) that were mapped and annotated on the chromosome. The data from K55-mop1 was made comparable with the other libraries by dividing the abundance of all small RNAs by 5.3, the average overall enrichment observed for miRNAs in the *mop1-1* mutant [92]. All the classes of repetitive elements analyzed expressed larger small RNA populations in the reproductive organs compared to leaves and showed a reduction in the *mop1-1* mutant, relative to wild type (Figure S13A and S13B). Unique small RNAs related to the *En-Spm* and *MuDR* families were significantly the most frequent among the DNA transposons, irrespective of the tissue and the genetic background. This is consistent with the finding that the *mop1* mutation can reverse the methylation status and silencing of *Mutator* elements in maize [96], probably via a reduction of the corresponding siRNA population [92]. Interestingly, the expected decrease of distinct signatures in K55-*mop1* compared to K55-wt was more remarkable for *MuDR* than for *En-Spm*, in particular when the total abundances were considered (77% reduction vs 50% for the two families, respectively). However, the size distributions of the two populations were very similar, both involving a majority of 24-mers in wild type that are expected to be reduced in a *mop1-1* background. Discrepancies between the small RNAs of the two varieties (K55 vs B73) also were observed. Ears from K55 showed slightly higher small RNA abundances for *MuDR* and *En-Spm* than the equivalent tissue of B73 (Figure 6C). Besides, a much more significant difference was observed in the opposite direction for the *hAT* family, which was more abundant in the small RNAs of B73 ears (2∶1). Further investigations are required to clarify to what extent this phenomenon is determined by different genetic backgrounds, environmental effects, or an imperfect correspondence between the developmental stages of the two samples.

**Table 3:** Total number of distinct small RNAs originating from different classes of sequences.

Library	DNA transposons	LTR retro-transposons	Centromeric and satellite sequences	Tandem repeats	Genes
**B73 - ZMA1**	1837	60154	578	36424	14161
**B73 - ZMA2**	4286	124401	986	71688	25281
**B73 - ZMA3**	4626	134070	970	79825	28035
**K55 -MOP1**	2633	219291	1342	145308	35234
**K55 - WT**	4899	153478	751	98543	36230
**Average**	3656	138279	925	86358	27788

LTR retrotransposons were the most prominent repeat class matched by small RNAs, consistent with their large proportion in the genome. Non-redundant small RNAs mapped within these elements were, when averaged across the libraries, 38-fold more numerous than those matching to DNA transposons. Accordingly, the sum of their abundances was 15-fold greater than the total abundance of DNA transposon-specific small RNAs, after a normalization based on the number of copies in the available contigs. Since the disparity can be only partially accounted for by the difference in unit length between the two classes of repetitive elements, this observation suggested a more pronounced tendency of LTR retrotransposon sequences to be processed into small RNAs, possibly because their replication cycle involves an RNA intermediate. One unexpected observation was that, in every sample, the LTR retrotransposons of the *Copia* superfamily in AR182 were represented by much fewer and less abundant small RNAs than the elements of the *Gypsy* superfamily (Figure S13). Because the difference in the total nucleotide length covered by the two superfamilies was negligible, this result suggests that *Copia* elements are less prone to provide templates for small RNA biogenesis. Nevertheless, considering the prominent role of siRNAs in the transcriptional silencing of transposable elements, the observed pattern of small RNA generation is not sufficient to explain the very low transcript level reported for most families of *Copia* LTR retrotransposons [97]. A total of ∼28,000 distinct signatures per library were found to match the gene space of AR182. This corresponded to 20% of the set of sRNAs originating from transposable elements, but within the overall length of the 544 genes analyzed, the density of distinct sRNAs was 10-fold larger compared to those in repeats and the mean total abundance per library (∼84,000 TPM) was not less than 25% of those from transposons. We noticed that many of the genic small RNAs matched an average of more than two genomic locations, possibly indicating either (1) sequence conservation of paralogs, or (2) mis-annotation of repetitive elements. A separate analysis of exons and introns revealed a strong bias for small RNAs accumulating in the latter, with introns having five times as many distinct small RNAs as exons, including a four-fold larger total abundance after correcting for hits to the contigs (Table S11). Further analysis demonstrated that 64% of the intronic small RNAs matched to identifiable repetitive elements.

The impact of repetitive elements on the small RNAs in proximity to gene promoters and trailers (upstream and downstream of annotated genes) also was analyzed. The upstream sequences of the 544 genes were investigated in 50 bp windows starting from the putative transcription start site. While the occurrence of TEs gradually increased from 2.7% to 12% between 1 and 200 bp upstream of the genes and again from 12% to 17% between 250 and 400 bp, the number of distinct signatures matching to the first region (1 to 200 bp) was limited and rapidly increased in the second genomic interval (250 to 400 bp). The same pattern was apparent and even more evident when the total abundances of the matching sRNAs were analyzed (Figure S14). Moreover, after correcting the abundances for the hits to the contigs, a comparison to a region further upstream revealed that the 1–200 bp interval was predominately matched by low-copy-number signatures. The analysis of downstream sequences showed a very similar profile, indicating a general paucity of small RNAs relative to the occurrence of TEs in the flanking regions next to the gene boundaries. However, this reduced set of small RNAs is only observable over a short distance both in 3′ gene trailers and 5′ gene promoters.

All small RNA data from this analysis can be accessed at http://mpss.udel.edu/maize/.

### Annotation comparison of AR182 to its corresponding region (AGP182) in B73 RefGen_v1

Consistent with the additional data used to construct the pseudomolecule (i.e. overlapping sequences, and ordering and manually orienting sequence contigs based on optical map evidence) and the degree of manual annotation/curation it received, AR182 exhibits significant improvement (see Materials and Methods) as compared to AGP182, the corresponding sequence in B73 RefGen_v1 [23], (Table 4). The total sequence contig number was reduced from 1170 to 907 whereas the average size of each contig increased from 18,923 bp to 23,860 bp. Similarly, the number of scaffolds was reduced from 544 in the highly automated AGP182 assembly to 440 in AR182, and the average scaffold size increased from 40,819 bp in AGP182 to 49,238 in AR182.

**Table 4:** Sequence and gene content comparison between AR182 and AGP182.

Feature	AR182	AGP182
**Size with Ns (bp)**	21,702,972	22,259,975
**Size without Ns (bp)**	21,640,322	22,140,315
**Number of contigs**	910	1170
**Unique sequences (bp)**	140,460	555,826
**Unique sequence percentage**	0.63%	2.50%
**Orientation-changed sequences (bp)**	2,192,652	2,288,766
**Identical sequences (bp)** a	19,307,210	19,295,723
**Gene models**	544	493
**Identical genes** a	304	305
**Overlapping genes**	125	122
**Unique genes**	115	63^b^

**a** number difference is due to unresolved tandem duplication.

**b** excluding 3 genes from contamination.

The use of the enhanced AR182 sequence led to slight but detectable differences in the annotation of repetitive elements compared to AGP182 (Table 1). While the identified coverage of all TE types was similar between AR182 and AGP182, they appeared less fragmented on AR182 in comparison to AGP182, a finding that is likely due to the improved assembly of AR182. Because the same databases were used to RepeatMask AR182 and AGP182, any difference between them can be attributed to the differences in the level of sequence assembly and improvement. For example, from the comparisons of nested TE insertions (Figure S1A versus Figure S1B and Figure S2A versus Figure S2B), more complete LTR elements could be detected on AR182. This more complete description of TEs will improve detection sensitivity and characterization of TEs in future projects, and by extension improve the specificity of gene annotations as well.

In the MGSP, the low-copy regions of the genome were finished at high quality. Indeed, with the exception of two 1-bp mismatches, and a 1-bp gap in three genes (highlighted in Table S8), the sequences of all predicted genes from AGP182 were identical to sequences present in AR182. These variations however had no effect on the three open reading frames and protein translations, except for a single amino acid substitution in gene ZmAcc7g20000928.

Although the current draft sequence is of tremendous utility to the maize/plant genetics research community as it stands today, like any genome sequence and annotation, it could be improved by the application of additional time, resources, new methods, technologies, analysis tools, etc. This manuscript attempts to quantify the benefits of doing so to a reasonable approximation for a small region of the maize genome. Our results demonstrate the feasibility of refining the B73 RefGen_v1 genome assembly by incorporating optical map, high-resolution genetic map, and comparative genomic data sets. Such improvements, along with those of gene and repeat annotation, will serve to promote future functional genomic and phylogenomic research in maize and other grasses.

## Materials and Methods

### Shotgun sequencing, assembly, and sequence improvement

BAC shotgun sequencing (176 BACs total) and finishing were performed using standard and previously published protocols [23],[98]. Each BAC received two 384-well paired end sequences, which resulted in ∼4–6× coverage, depending on the BAC insert size.

Sequence data for each BAC was assembled, confirmed using BAC end sequence, checked for minimum coverage standards, and sent for automated sequence improvement. Prior to sequence improvement, fosmid end sequences [22],[23], were added to the assemblies to enhance order and orientation. Consensus sequence data were evaluated by K-mer analysis [39] to determine repeat content. Automated improvement involved directed sequencing reactions across all gaps and low quality areas within non-repetitive regions of the sequence. Following automated sequence improvement, additional data, in the form of cDNA sequences and sequences from subtractive libraries using methyl-filtered DNA and high *C_o_t* techniques [7] available from GenBank, were incorporated into the assemblies. Manual improvement was performed on non-repetitive regions only, using guidelines established by the MGSC (see supplemental material in [23]). Improved sequence was submitted to GenBank as phase-I improved (HTGS_IMPROVED).

### Pseudomolecule construction

Trace files from the 176 BAC shotgun sequencing projects (Table S2) were downloaded from the NCBI Trace archive. Trace files from 5–8 BACs were pooled and assembled with Phrap (http://phrap.org). Sequence contigs in each assembly were divided into different groups according to their BAC origin. Based on their positions in the physical map, BESs of related BACs were used to set the order and orientation of these sequence contigs. Within each BAC sequence, paired-end sequences were used to order and orient two contigs (>9 reads and >2 kb). Sequences from each assembly were exported to the ALL_AGI_CTG_SEQ database. Concurrently, sequences from the same 176 BACs from NCBI CoreNucleotide database were downloaded. These NCBI sequences were split into pieces according to gaps (100 Ns) and defined as the Genome Center at Washington University (GCWU) sequences. A Mega BLAST search (identity >98%) was then run using each GCWU sequence as a query against the ALL_AGI_CTG_SEQ database. If AGI sequences were found, the GCWU sequences were ordered and oriented according to the AGI assembly and were recorded in the final pseudomolecule as a fragment. If no AGI sequence was found, then the sequence was disregarded. The pseuodomolecule was further adjusted by comparing it with the maize Optical Map [40]. Regions of disparity called by the Optical Map were manually curated and modified for accurate ordering and orientation and contaminated sequence removal. Extra sequences present in the pseudomolecule, but not on the Optical Map, were used as query sequences to search against the maize genome sequence using BLASTN. In most cases, these sequences hit other genomic regions, indicating slight sequence contamination, and were removed from the pseudomolecule. Although maize-rice synteny was also used to order and/or orient some fragments, these refinements were then validated by comparisons to the Optical Map. Finally, gaps were filled between sequence fragments by using a series of Ns (50 Ns to fill gaps between ordered and oriented contigs; a pair of 60 Ns to tag internal fragments that were ordered but not oriented; a pair of 100 Ns to tag internal fragments that were neither ordered nor oriented; and 80 Ns inside a pair of 100 Ns to connect two fragments (or blocks) with unknown order and orientation). Comparisons with the Optical Map showed that ∼600 kb of sequence was missing from the AR182 assembly, stemming largely from misassembly of nearly identical retrotransposon LTRs. The sequence order could not be determined for two large nested retrotransposon insertion complexes (regions from 14 to 14.3 Mb, and 19.0 to 19.4 Mb); however, only a single gene in each region was identified (Figure 2). Otherwise, gap sizes should be minimal (<500 bp in size based on the resolution of the Optical Map [40]). In total, the AR182 pseudomolecule contained 21,702,972 bp of sequence, composed of 907 sequence fragments and 906 gaps.

### Repeat analysis

#### LTR retrotransposon discovery and description

Two *de novo* retroelement identification programs, LTR_STRUC [99] and LTR_SEQ [100] were used in an attempt to capture all of the intact LTR retrotransposons on the pseudomolecule. Both programs were run under their respective default parameter settings. These programs output the start and end co-ordinates of each full-length element and structure information such as the target site duplication (TSD), primer binding site (PBS), and TG..CA motif that terminates each LTR. Additional BLAST-based and hand annotations were performed to validate the authenticity of each of these predictions. The results of the analysis yielded a total of 476 full-length elements. This included 93 elements that LTR_STRUC alone detected, 165 elements that LTR_SEQ alone detected, 217 that both programs detected identically, and 1 element that both programs detected with a substantial overlap. The elements accounted for an aggregate of 4.7 Mb (21.7% of the pseudomolecule).

Previous work demonstrated that many of the LTR retrotransposons in plants can be highly variable in structure, ranging from intact (both LTRs present along with internal sequence) to fragmented (one LTR plus internal sequence), to being represented by a single solo-LTR [101],[102]. Thus, to uncover all LTR retrotransposons on the pseudomolecule, a homology-based identification program, RepeatMasker (vers. 3.15; [103]), was used to comprehensively annotate all LTR retrotransposons using a locally curated database (P. SanMiguel, pers. comm.). To determine the relative structural state of these annotations, BLASTN searches (e^−10^) were performed with a database of full-length or solo-LTRs separately after masking out the positions of the structurally-determined full-length elements. A representative element from each of the 109 families identified in the RepeatMasker search was included in each database. The results of these two BLAST analyses were aligned and the relative number and length of fragments versus solo-LTRs was determined; an element was considered a fragment if it exhibited homology to a sequence from the solo-LTR database and also exhibited a longer alignment to an element from the full-length database of elements. Further, an element was considered a solo LTR if it exhibited a significant hit to an element from the solo-LTR database and was the same length as a significant hit to an element from the full-length LTR database. We used TE nest [104] to graphically view nested TE insertion.

#### Discovery and description of LINEs and SINEs

Other retroelements were identified through both structural and homology-based searches. LINE elements were uncovered by performing a homology-based screen using BLASTX (e^−10^) to a protein database (PTREP). The annotations of LTR retrotransposons, LINEs and SINEs were overlayed in the visual curation program Apollo (vers. 1.65; [105]) and manually screened for overlaps or miss-calls between element types. The structure-based search process for SINE was described in Baucom et al [45].

#### Gene acquisition by retroelements

To determine if *Z. mays* genes were found within LTR retrotransposons, both the LTR retrotransposon-masked and unmasked versions of the pseudomolecule were screened for host genes using a BLASTN search (e^−10^) to a database of EST sequences from *Arabidopsis thaliana* (Release 13.0, The Gene Index Project). Significant hits to regions from the LTR retrotransposon-masked pseudomolecule were removed from the BLAST results using the unmasked pseudomolecule to remove genes that were not found within LTR retrotransposon regions. Significant alignments between *A. thaliana* EST sequences then were parsed from the pseudomolecule and screened for homology to a database of LTR retrotransposon genes (*gag*, *rnaseh*, *integrase* and *reverse transcriptase*) curated from PFAM [106]. Sequences that did not exhibit similarity to LTR retrotransposon genes were then screened further by performing a BLASTN (e^−1^) to NCBI's non-redundant database, and sequences that did not exhibit homology to a repetitive element were considered to be host genes found within LTR retrotransposons.

#### DNA element discovery and description

A library of classical DNA TEs (CACTA, *hAT*, MULE, *Mariner*-like elements and *PIF* like elements was constructed by structural criteria and by repetitive features as described [23]. Each element sequence in the library was considered a distinct family. The sequences in the library were used to mask AR182 and AGP182 and the output of RepeatMasker was used for estimation of copy number and coverage of each superfamily. Redundant matches in the output were eliminated by excluding the shorter match (for copy number calculation). That is, if two elements matched the same region and the sequences overlapped by 90% or more, the shorter match was removed. If an element in the genomic sequence matched an exemplar over the entire sequence, or if the truncation was less than 20 bp on each end, this element was considered to be an intact element. Otherwise it was considered as a truncated element or half of a copy. Fragmented elements that lack both ends (truncated more than 20 bp on both ends) were not included in copy number estimation. The coverage of TEs was estimated as the total sequence masked by each superfamily with overlapping regions only calculated once. The number of families of each superfamily in the sequenced regions was determined as the number of families (from the TE library) present on the RepeatMasker output. If a single element matched two or more families on the RepeatMasker output, the one with longest match was considered to be the family this individual element belongs to. *Helitrons* were identified by structural criteria using a bioinformatic tool [107]. The program searches for *Helitron* 3′ end structures, and then aligns any cases where the same structure is found more than once. If this alignment indicates precisely appropriate boundaries, then the element is judged to be a *Helitron*. Candidate autonomous *Helitrons* were found by searching for encoded rolling-circle-replication-related proteins (Rep/helicase and RPA). Gene fragments acquired by *Helitrons* were identified by a BLASTX search against the NCBI nr database, with a maximum Expect value of e^−10^ required to a genome other than maize.

#### Mathematically-defined repeats

Maize whole-genome shotgun reads generated by the Joint Genome Institute were downloaded from the NCBI Trace Archive and clipped to remove vector sequences using cross_match (http://www.phrap.org/) and the NCBI UniVec database (http://www.ncbi.nlm.nih.gov/VecScreen/UniVec.html). These were used to build a suffix array index of all 20-mer sequences using Vmatch. Maize BACs were queried against the index to determine the relative frequency of the 20-mers initiated at each nucleotide position along the BAC sequence [39].

### Gene annotation

Repetitive elements were masked by RepeatMasker [103] using version 4.3 of the MIPS REdat database of plant repeats [44] and TE exemplar databases [45]. Protein-coding genes were annotated using a modification of the Ensembl evidence-based gene-build pipeline [66],[67]. The following available evidence was used: 11,742 cDNAs from the maize full-length cDNA sequencing project [108], 359,942 Swiss-Prot proteins from all species, 62,242 GenBank proteins from plant species, 1,462,607 ESTs and 18,181 other mRNAs from maize, 1,217,859 ESTs and 72,919 other mRNAs from rice, 2,448,641 ESTs and 14,015 other mRNAs from other monocot species. Putative genes were filtered using a minimum translation length of 30 amino acids. *Ab initio* prediction by Fgenesh [109] was used to supplement evidence-based models. Where a Fgenesh prediction overlapped with a partial evidence-based model (i.e. those lacking a start or stop codon) the models were combined to extend the coding sequence. Fgenesh models were included “as is” where no overlap was found with an evidence-based model. Resultant models were further screened for transposable elements by BLASTP alignment to NCBI GenPept and comparison of aligned subjects to a curated list of transposable elements derived from the same database. Misannotations caused by fusion of separate genes into one model or splitting of genes into multiple models were detected by TBLASTN alignment using rice or sorghum proteins as the query. Such models were reannotated using GeneMark [110]. For the additional evidence of the 115 AR182-unique genes, we used the most recent (up to July 17, 2009) EST/fl-cDNA and nr database from GenBank, the MSU rice annotation 6.0 (http://rice.plantbiology.msu.edu/), and the JGI sorghum genome annotation (http://genome.jgi-psf.org/Sorbi1/Sorbi1.home.html). DNA/amino acid sequences of the 115 genes were used as queries against the above databases in either BLASTn or tBLASTn searches.

### Identification of synteny and putative orthologs

All sequences in this analysis were masked as above prior to alignment with BLASTZ [82] and SyMAP (Synteny Mapping and Analysis Program;[83]). For *Oryza sativa* ssp. *japonica* we used the TIGR release 5 assembly [111]. For *Sorghum bicolor* we used the Sorbi1 assembly [71]. To identify the maize homeologous region we used physically-anchored BAC sequences and annotations from release 4a.53 of the Maize Genome Project [23]. To reduce the effects of overlapping BAC's, the annotated genes were screened for redundancy. The rice-only hypothetical genes (totaling 168 genes) and sorghum low-confidence genes (175 genes) were excluded in the final statistics due to the high potential of annotation error. Syntenic regions were defined as maximally-scoring, colinear chains as described [112]. Putative orthologs within these regions were identified as best reciprocal hits using BLASTP. Additional confidence in ortholog assignment was provided by filtering for colinearity. Reciprocal best hits were deemed colinear if separated by no more than 500 kb in either of the genomes being compared. This method is conservative since only a subset of lineage-specific duplications in the region can have a reciprocal best hit. In addition, it should be noted that this method would not distinguish misassignment due to reciprocal loss of adjacent paralogous genes [21].

TBLASTN was used to search of duplicated genes in the maize Chr5-related region using the AR182 set of 544 genes as queries against the Chr5-related BAC sequences at a cutoff of e-^10^. Manual inspection was performed to ensure gene colinearity.

### CDS feature mapping

Exons within coding sequences were aligned in a pairwise manner between othologous genes using BLASTN. Positive mappings were assigned to those exons having an e-value threshold of 1e^−5^, at least 80% identity, and matching orientation. Intron mappings were assigned when both flanking exons of one gene mapped to the flanking exons of its ortholog, and such exons occurred in the same order relative to their respective coding sequences. Eighty-eight percent of maize exons mapped to rice and 94% mapped to sorghum. Identical exon/intron structures were found in 51% of rice-maize orthologs and in 66% of sorghum-maize orthologs. Overall, we recovered 1,268 intron pairs between maize and sorghum and 1,114 pairs between maize and rice.

### Computational identification of putative miRNA paralogs in genomic sequences

Annotation of miRNA genes on AR182 was performed as identified as described in [74]. Annotations were similarly performed on BAC clone sequences present in the maize chromosome 5 homeologous region [23] For rice we used TIGR Release 5 [111] and for sorghum we used the Sorbi1 assembly [71].

### Ka/Ks analysis

Amino acid sequences were translated from coding sequences and aligned in a pairwise fashion using CLUSTALW version 1.83 [113]. Alignments were mapped to coding sequence coordinates and alignment gaps were removed using methods available in the BioPerl toolkit [114]. Rates of non-synomous (Ka) and synonymous (Ks) substitutions were estimated with codeml of the PAML package version 3.15 [115] using the F3X4 codon frequency model. Differences in the distributions of divergence rates were evaluated for significance using the non-parametric Mann-Whitney test [89] as implemented in the MedCalc statistical software package (MedCalc Software, version 9.3.8.0, Mariakerke, Belgium).

### Small RNAs preparation and sequencing

All the small RNA libraries were generated by ligation of the small RNA fraction to 5′ and 3′ adaptors followed by RT-PCR amplification and sequencing with Illumina's SBS technology. While the sequencing of K55-wt and K55-mop1 libraries was performed on multiple flow cell channels, yielding 5.6 and 7.2 million signatures respectively, the libraries from the B73 genotype were processed on single channels, resulting in an average of 4.2 million signatures each. The abundance of each sequence was normalized to 5 million [units of transcripts per 5 million (TP5M)] in all the libraries.

## Supporting Information

Figure S1 An example of nested transposable element insertion. This figure was generated by the TEnest program [104]. (A) AR182 (from 5,063,196 to 6,038,602; (B) AGP182, the AR182 corresponding region in B73RefGen_v1.
(1.25 MB PPT)

Figure S2 An example of nested transposable element insertions. This figure was generated by the TEnest program [104]. (A) AR182 (from 17,063,504 to 18,059,878; (B) AGP182, the AR182 corresponding region in B73RefGen_v1.
(1.44 MB PPT)

Figure S3 DNA transposon and gene distribution along AR182. The distribution was constructed based on nucleotide length of the related TE in 100-kb sliding windows. The numbers at the left vertical axis represent the nucleotide length of related TE classifications. The numbers in the right axis are the gene number counts.
(0.09 MB PPT)

Figure S4 The distribution of numbers of intergenic spaces and their sizes in AR182. The spaces less that zero kb indicated gene overlap.
(0.15 MB PPT)

Figure S5 Correlation of intron sizes among maize, rice, and sorghum. (A) Maize-sorghum orthologs; (B) Maize-rice orthologs. Pearson's correlation coefficient and 95% confidence interval is shown for intron lengths <1 kb (inset). Above 1 kb, maize intron lengths are notably elevated relative to their ortholog.
(0.12 MB PPT)

Figure S6 Intron size distribution among maize, rice, and sorghum.
(0.06 MB PPT)

Figure S7 Intron length discrepancies among maize, sorghum, and rice in the AR182 homologous regions.
(0.06 MB PPT)

Figure S8 Relationship between sequence length differences in ortholologous introns and the presence of repetitive sequences. Intron length differences are calculated as (length of the maize intron)-(length of the sorghum intron), such that negative values occur when the maize intron is shorter than its ortholog. Each intron length difference is plotted against repetitive content.
(0.08 MB PPT)

Figure S9 Example of recent insertions of LTR retrotransposons into the intron of an active maize gene. The depicted gene (ZmAcc7g20001011) encodes a transcript corresponding to the full-length cDNA clone ZM_BFb0042A02 (gb accession BT041740), whose translation product is homologous to members of the haloacid dehalogenase superfamily. Two retrotransposons are shown inserted in a nested fashion into the fourth intron. As determined using K-mer and TEnest software [39],[104], the first was classified as a member of the machiavelli family (Copia superfamily), with a date of insertion estimated at 615 thousand years ago. A second insertion was classified as a member of the jaws family (Gypsy superfamily). Although LTR sequences flank jaws, these were classified as solo LTRs. As shown by TBLASTN alignments, all exons are conserved with corresponding orthologs in sorghum and rice, including those that flank the fourth intron. The exon-intron structure is conserved amongst the three orthologous genes, but whereas the the fourth intron is greater than 12 kb in maize, the corresponding introns are only 647 bp and 264 bp in sorghum and rice respectively. DNA-based alignments (BLASTZ/ChainNet) showed extensive coverage of both exons and introns with syntenic regions of rice and sorghum, but retrotransposon sequences did not align.
(0.06 MB PPT)

Figure S10 An example of recent maize duplication and its synteny with rice. This SyMAP generated figure is the synteny analysis using the maize physical map saturated with genetic markers and rice pseudomolecules. In the middle is rice sequence and the left is the maize Chr4 region in this study, and on the right is the maize region from contig 250 to 254 on Chr5.
(0.64 MB PPT)

Figure S11 Direct comparison between maize AR182 and its orthologous sorghum pseudomolecules. SyMAP 3 [83] was used to perform the maize pseudomolecule (left) to sorghum pseudomolecule (right) comparisons.
(0.34 MB PPT)

Figure S12 Small RNA size distributions in the analyzed libraries. (A) Number of distinct small RNAs in different RNA size categories. (B) Total Number of small RNAs in different RNA size categories.
(0.10 MB PPT)

Figure S13 Small RNAs originating from LTR-retrotransposons. (A) Number of distinct LTR-retrotransposon-related small RNAs in different genetic backgrounds. (B) Total Number of LTR-retrotransposon-related small RNAs in different genetic backgrounds.
(0.10 MB PPT)

Figure S14 Distribution of repetitive elements and small RNAs proximal to gene sequences. (A) Occurrence of repetitive sequences in the upstream of genes (in percentage); (B) Occurrence of repetitive sequences in the downstream of genes (in percentage); (C) Total number of small RNAs in the upstream of genes; (D) Total number of small RNAs in the down stream of genes.
(0.14 MB PPT)

Table S1 Genes and QTL mapped in AR182.
(0.03 MB XLS)

Table S2 The sequenced BAC clone list in AR182.
(0.11 MB XLS)

Table S3 Genetic markers in AR182.
(0.04 MB XLS)

Table S4 Physical to genetic ratios in AR182.
(0.02 MB XLS)

Table S5 General characteristics of the LTR elements in AR182.
(0.02 MB XLS)

Table S6 The general properties of LINES and SINES in AR182.
(0.07 MB XLS)

Table S7 Gene fragments captured by TE elements in AR182.
(0.05 MB XLS)

Table S8 Annotated gene list in AR182.
(0.06 MB XLS)

Table S9 Clones list of the AR182 homologous region in Chr5.
(0.03 MB XLS)

Table S10 Summary of small RNAs mapping on AR182.
(0.01 MB XLS)

Table S11 Summary of small RNAs mapping on exons and introns in AR182.
(0.01 MB XLS)
