# Supplementary figures and images for: Detailed Analysis of a Contiguous 22-Mb Region of the Maize Genome
Source: PLoS Genet. 2009 Nov 20;5(11):e1000728. doi: 10.1371/journal.pgen.1000728 (PMC2773423; doi:10.1371/journal.pgen.1000728)

## Slide 1
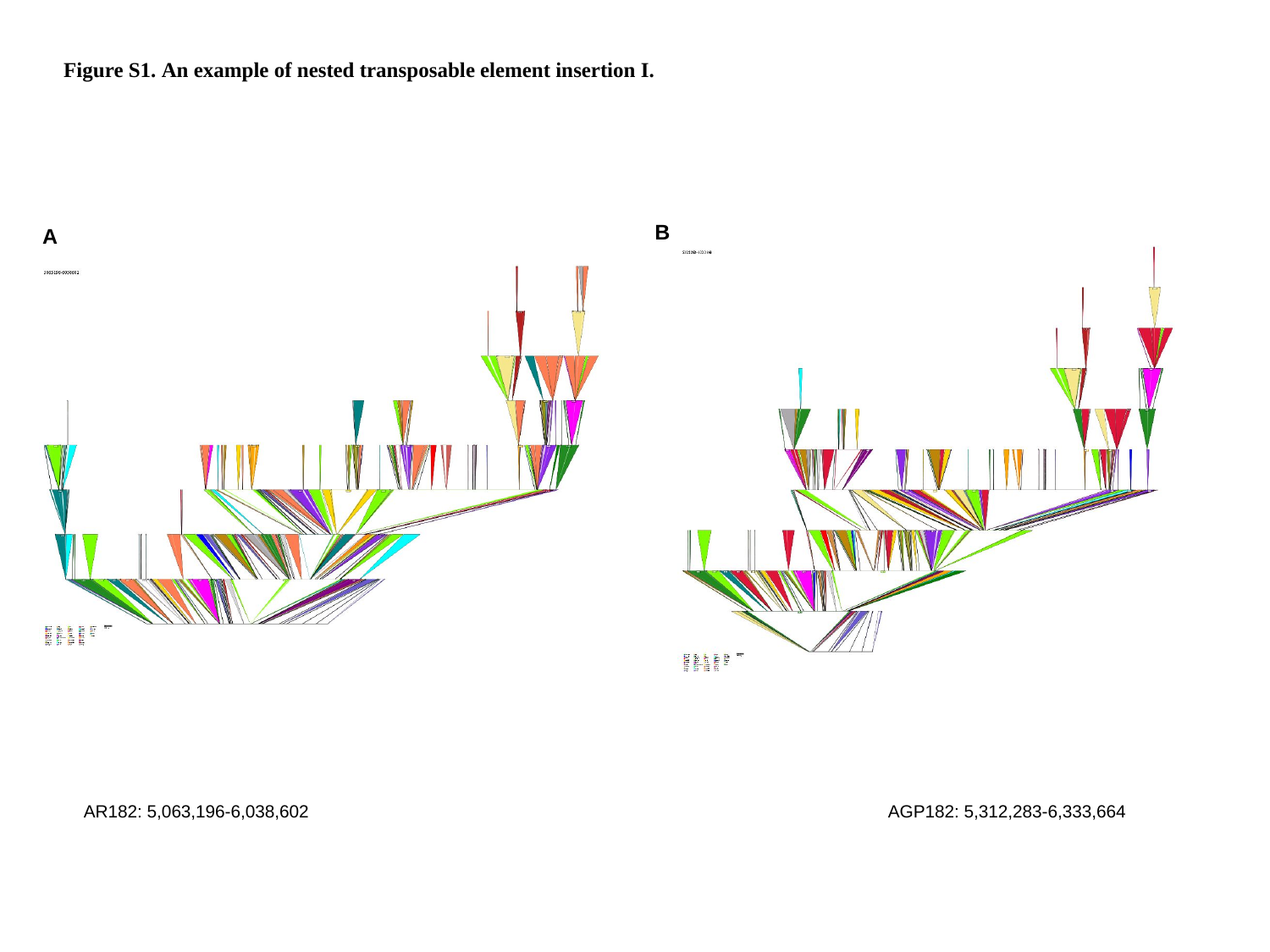

Figure S1. An example of nested transposable element insertion I.
B
A
AR182: 5,063,196-6,038,602
AGP182: 5,312,283-6,333,664

Supplement: Figure S1 — An example of nested transposable element insertion. This figure was generated by the TEnest program [104]. (A) AR182 (from 5,063,196 to 6,038,602; (B) AGP182, the AR182 corresponding region in B73RefGen_v1. (1.25 MB PPT) [file pgen.1000728.s001.ppt]

## Slide 1
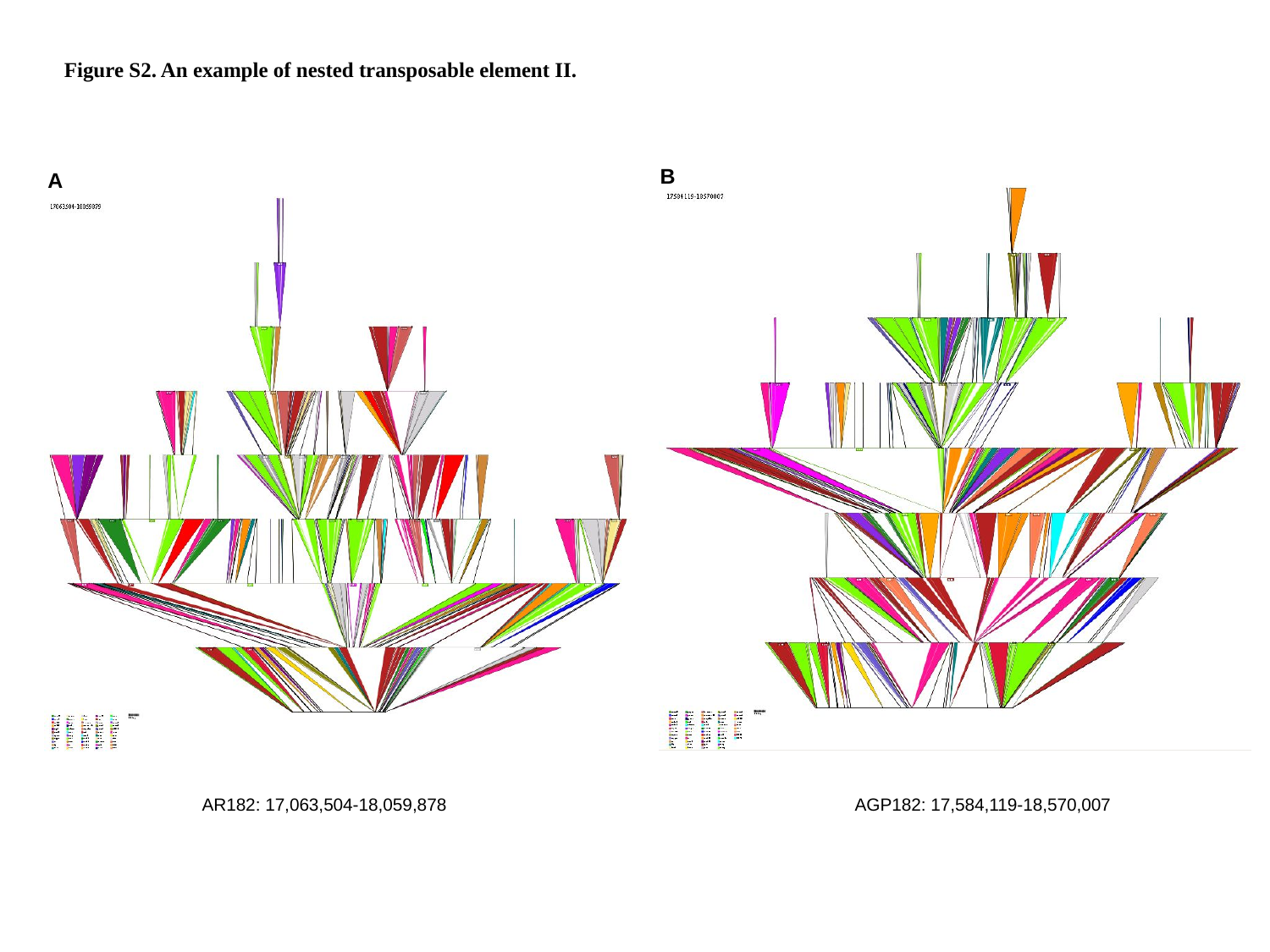

Figure S2. An example of nested transposable element II.
B
A
AR182: 17,063,504-18,059,878
AGP182: 17,584,119-18,570,007

Supplement: Figure S2 — An example of nested transposable element insertions. This figure was generated by the TEnest program [104]. (A) AR182 (from 17,063,504 to 18,059,878; (B) AGP182, the AR182 corresponding region in B73RefGen_v1. (1.44 MB PPT) [file pgen.1000728.s002.ppt]

## Slide 1
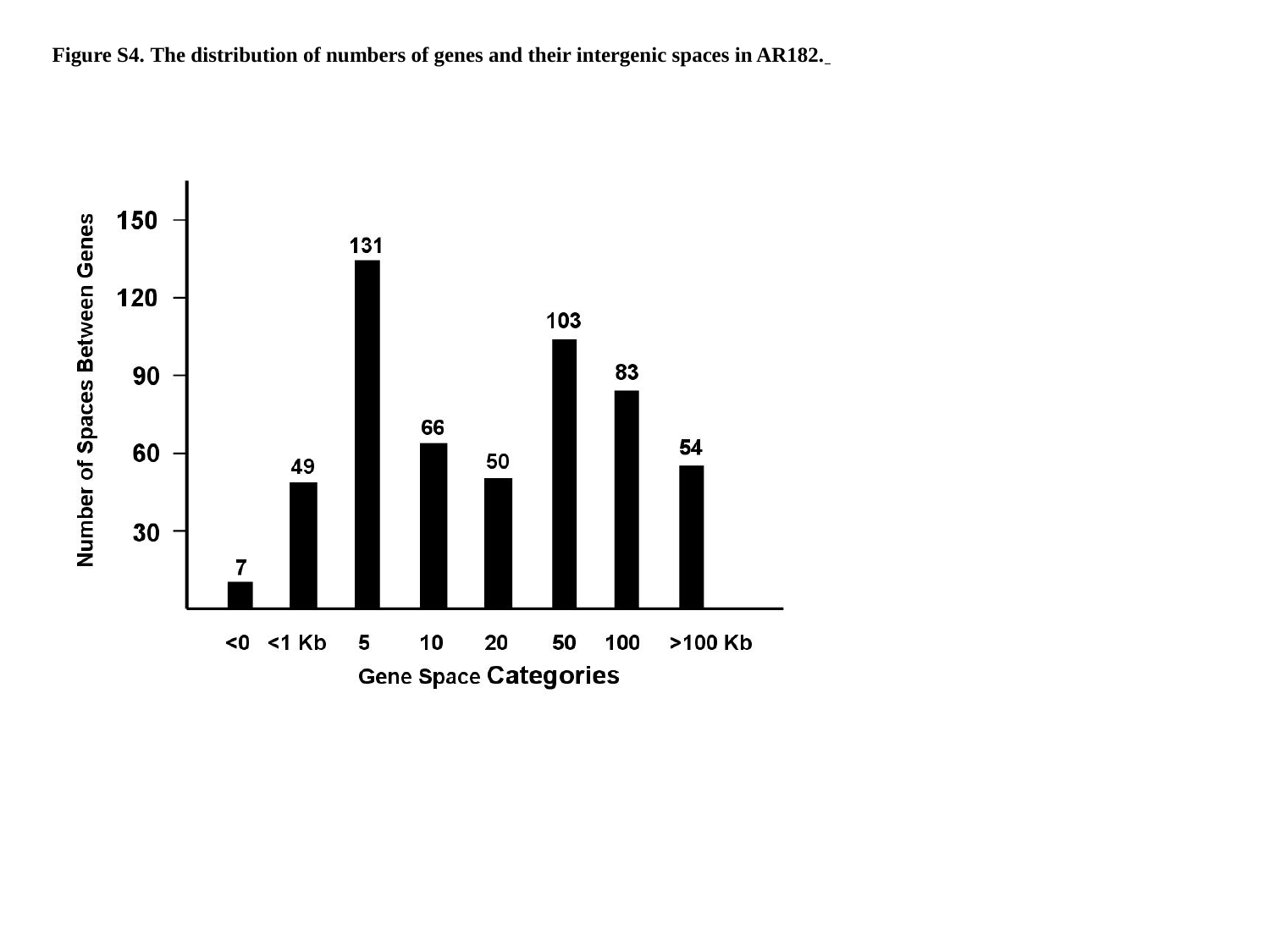

Figure S4. The distribution of numbers of genes and their intergenic spaces in AR182.

Supplement: Figure S4 — The distribution of numbers of intergenic spaces and their sizes in AR182. The spaces less that zero kb indicated gene overlap. (0.15 MB PPT) [file pgen.1000728.s004.ppt]

## Slide 1
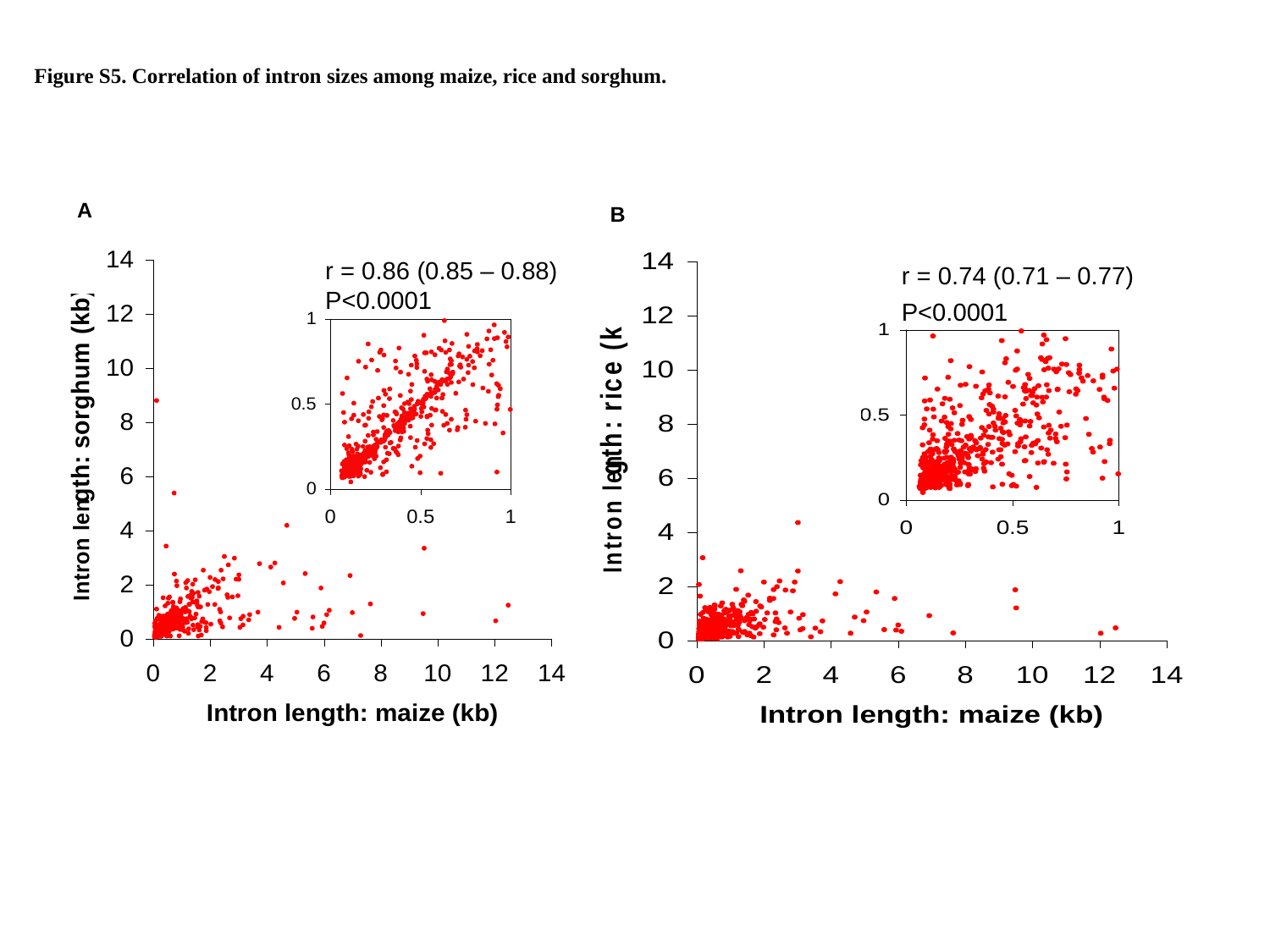

Figure S5. Correlation of intron sizes among maize, rice and sorghum.
A
B
r = 0.86 (0.85 – 0.88)
P<0.0001
r = 0.74 (0.71 – 0.77)
P<0.0001

Supplement: Figure S5 — Correlation of intron sizes among maize, rice, and sorghum. (A) Maize-sorghum orthologs; (B) Maize-rice orthologs. Pearson's correlation coefficient and 95% confidence interval is shown for intron lengths <1 kb (inset). Above 1 kb, maize intron lengths are notably elevated relative to their ortholog. (0.12 MB PPT) [file pgen.1000728.s005.ppt]

## Slide 1
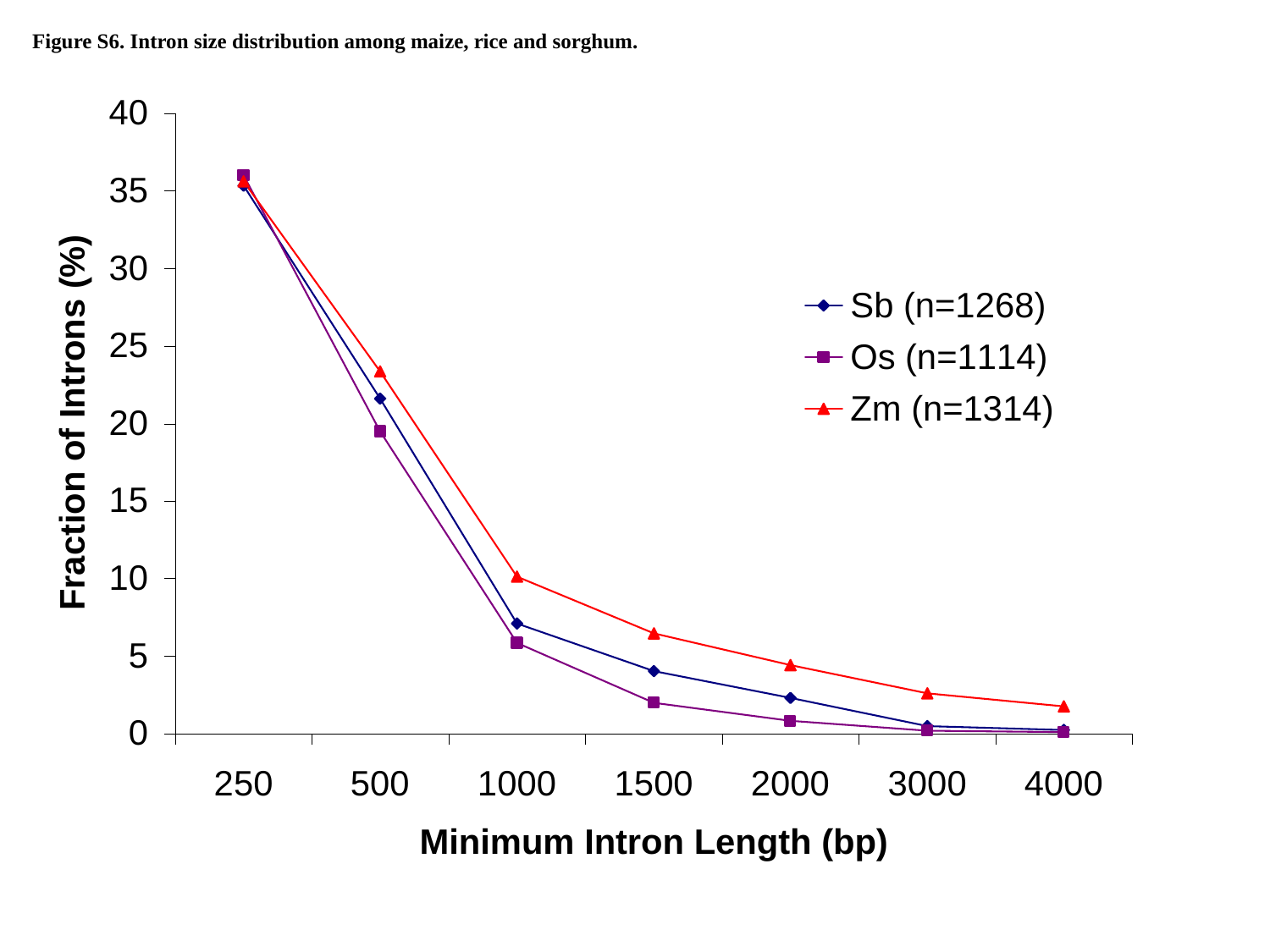

Figure S6. Intron size distribution among maize, rice and sorghum.

Supplement: Figure S6 — Intron size distribution among maize, rice, and sorghum. (0.06 MB PPT) [file pgen.1000728.s006.ppt]

## Slide 1
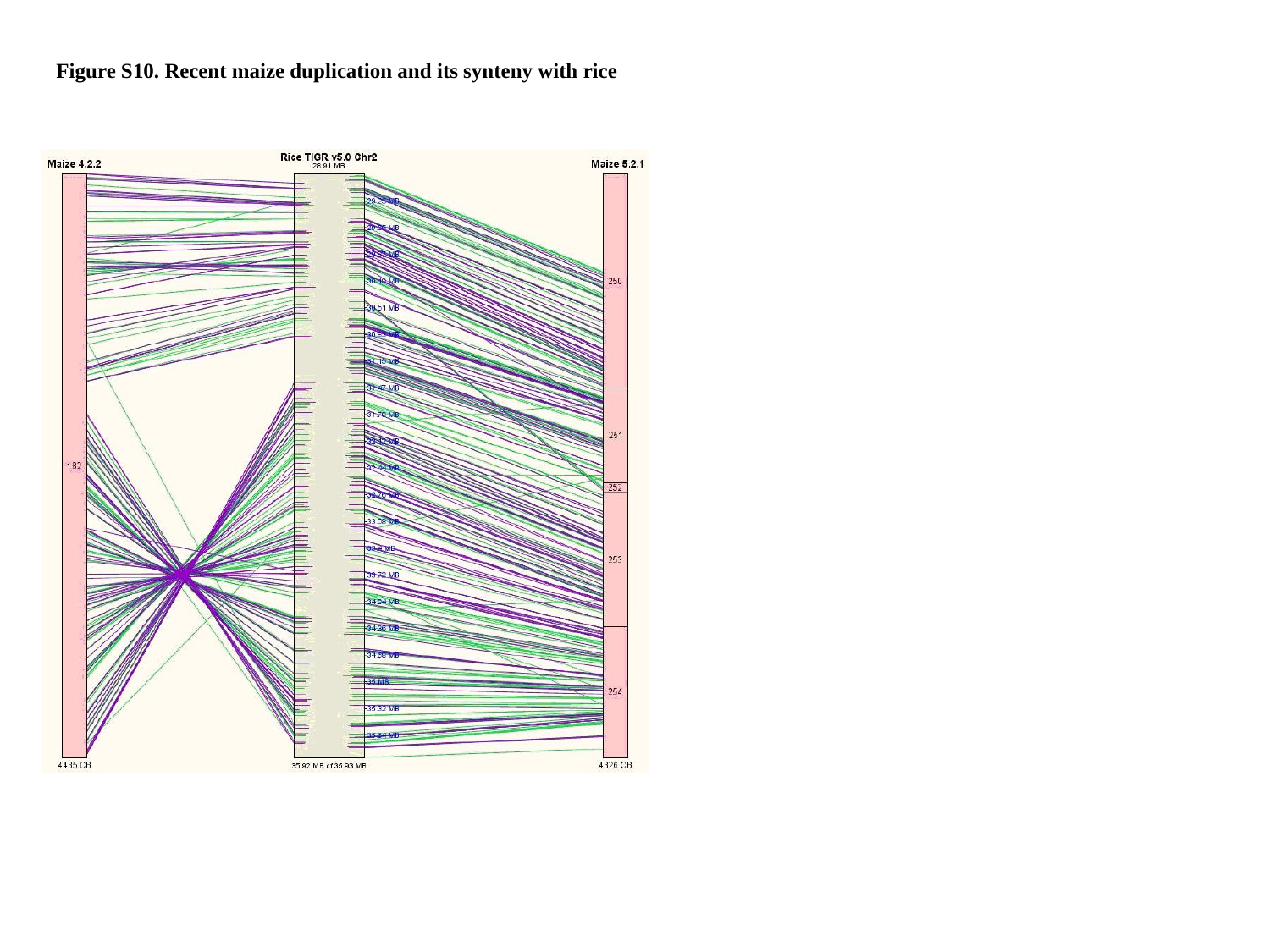

Figure S10. Recent maize duplication and its synteny with rice

Supplement: Figure S10 — An example of recent maize duplication and its synteny with rice. This SyMAP generated figure is the synteny analysis using the maize physical map saturated with genetic markers and rice pseudomolecules. In the middle is rice sequence and the left is the maize Chr4 region in this study, and on the right is the maize region from contig 250 to 254 on Chr5. (0.64 MB PPT) [file pgen.1000728.s010.ppt]

## Slide 1
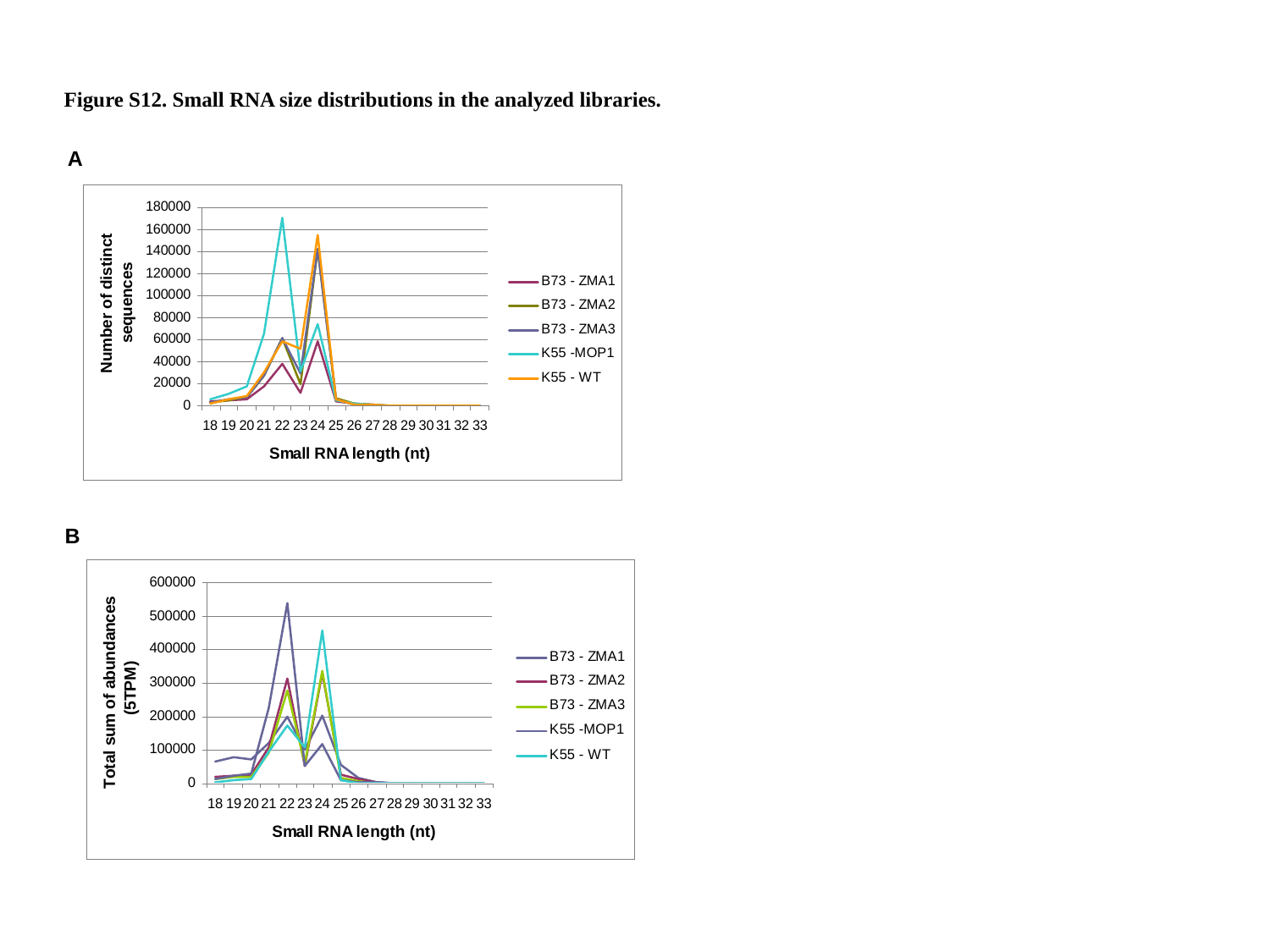

| Figure S12. Small RNA size distributions in the analyzed libraries. |
| --- |
A
B

Supplement: Figure S12 — Small RNA size distributions in the analyzed libraries. (A) Number of distinct small RNAs in different RNA size categories. (B) Total Number of small RNAs in different RNA size categories. (0.10 MB PPT) [file pgen.1000728.s012.ppt]

## Slide 1
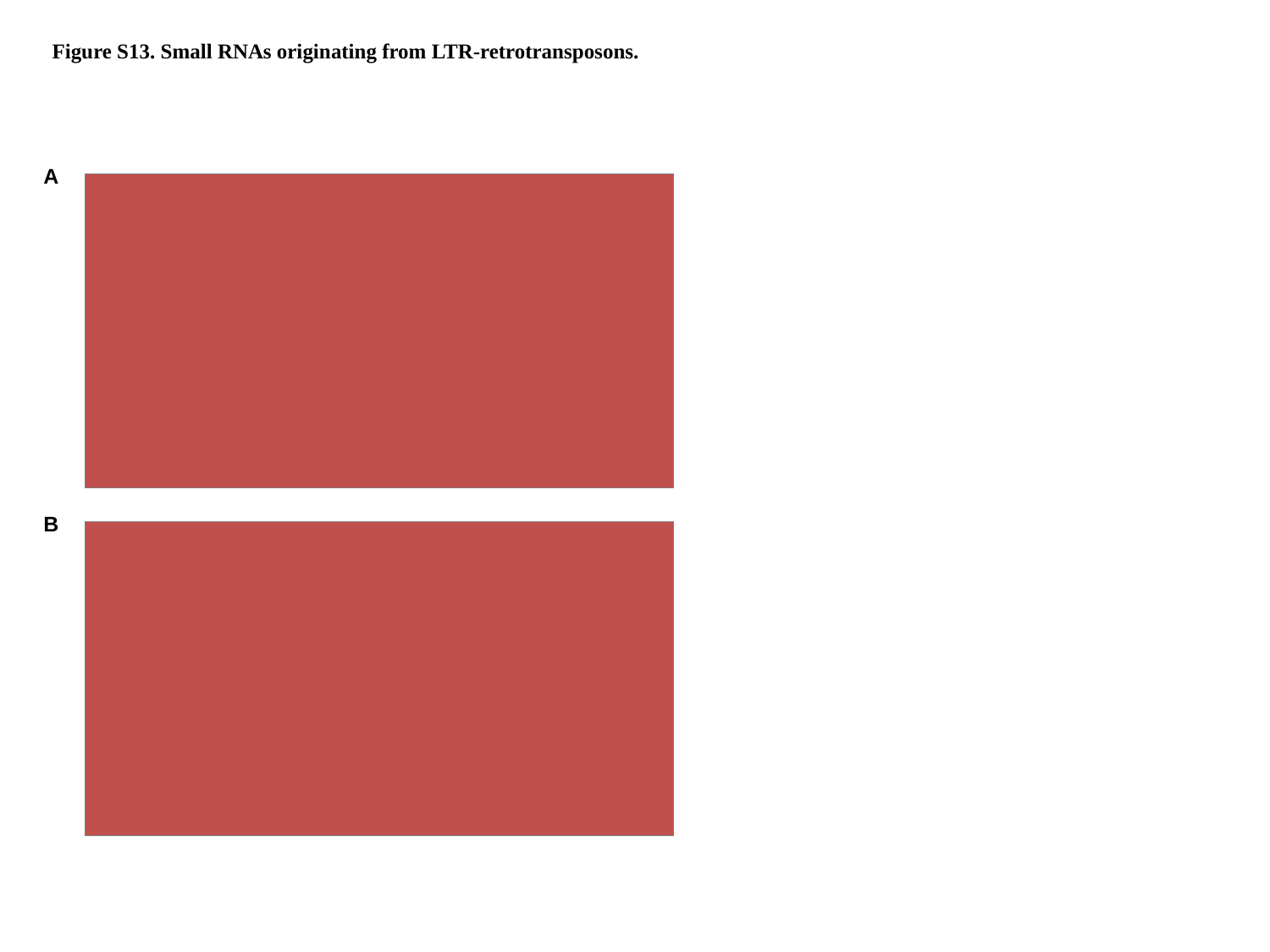

| Figure S13. Small RNAs originating from LTR-retrotransposons. |
| --- |
A
B

Supplement: Figure S13 — Small RNAs originating from LTR-retrotransposons. (A) Number of distinct LTR-retrotransposon-related small RNAs in different genetic backgrounds. (B) Total Number of LTR-retrotransposon-related small RNAs in different genetic backgrounds. (0.10 MB PPT) [file pgen.1000728.s013.ppt]
