# Supplementary material for: Detailed Analysis of a Contiguous 22-Mb Region of the Maize Genome
Source: PLoS Genet. 2009 Nov 20;5(11):e1000728. doi: 10.1371/journal.pgen.1000728 (PMC2773423; doi:10.1371/journal.pgen.1000728)
Supplement: Figure S3 — DNA transposon and gene distribution along AR182. The distribution was constructed based on nucleotide length of the related TE in 100-kb sliding windows. The numbers at the left vertical axis represent the nucleotide length of related TE classifications. The numbers in the right axis are the gene number counts. (0.09 MB PPT) [file pgen.1000728.s003.ppt]

## Slide 1
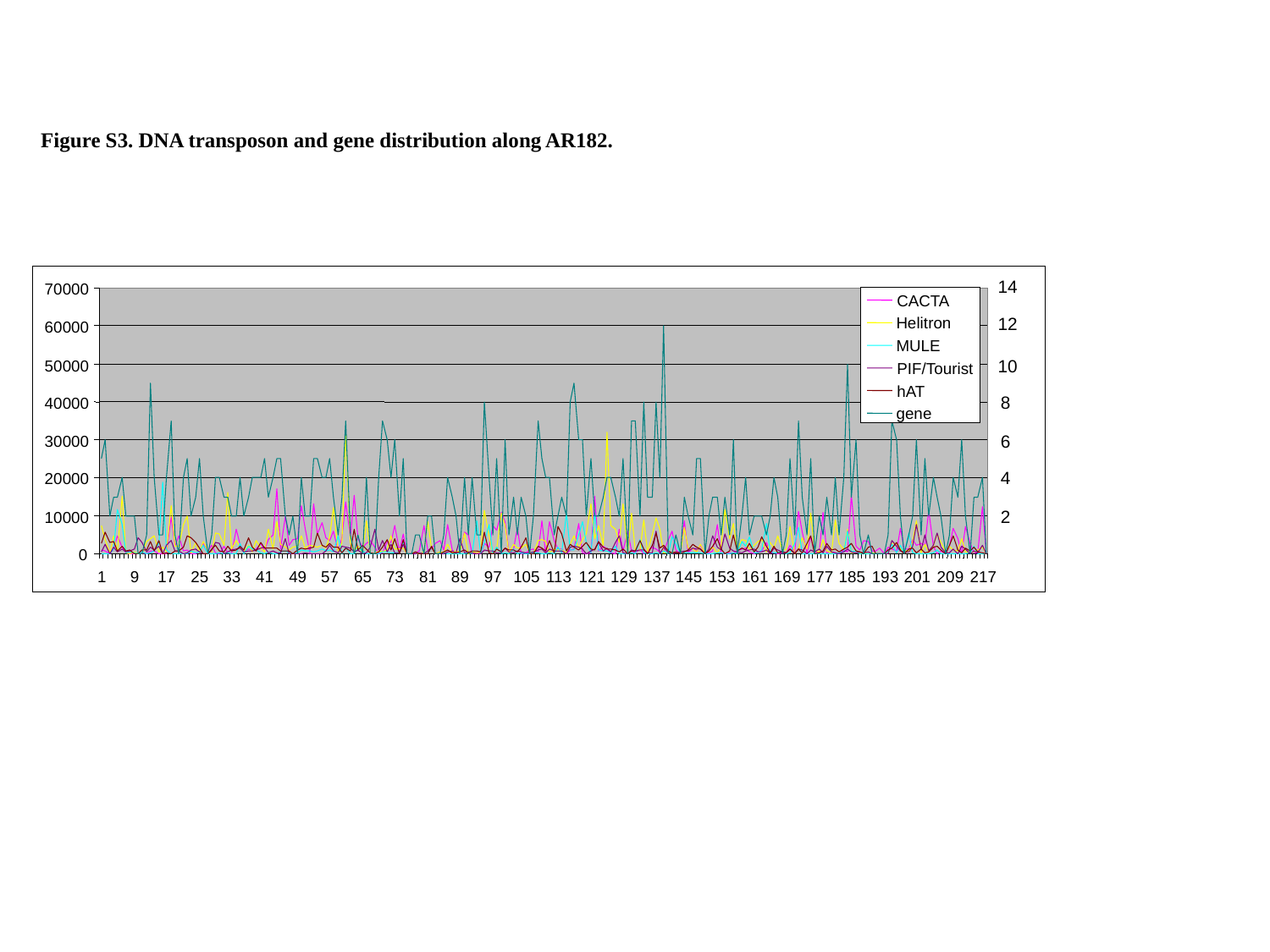

Figure S3. DNA transposon and gene distribution along AR182.
14
70000
CACTA
Helitron
MULE
PIF/Tourist
hAT
gene
12
60000
10
50000
8
40000
6
30000
4
20000
2
10000
0
1
9
17
25
33
41
49
57
65
73
81
89
97
105
113
121
129
137
145
153
161
169
177
185
193
201
209
217
