# Supplementary material for: Detailed Analysis of a Contiguous 22-Mb Region of the Maize Genome
Source: PLoS Genet. 2009 Nov 20;5(11):e1000728. doi: 10.1371/journal.pgen.1000728 (PMC2773423; doi:10.1371/journal.pgen.1000728)
Supplement: Figure S7 — Intron length discrepancies among maize, sorghum, and rice in the AR182 homologous regions. (0.06 MB PPT) [file pgen.1000728.s007.ppt]

## Slide 1
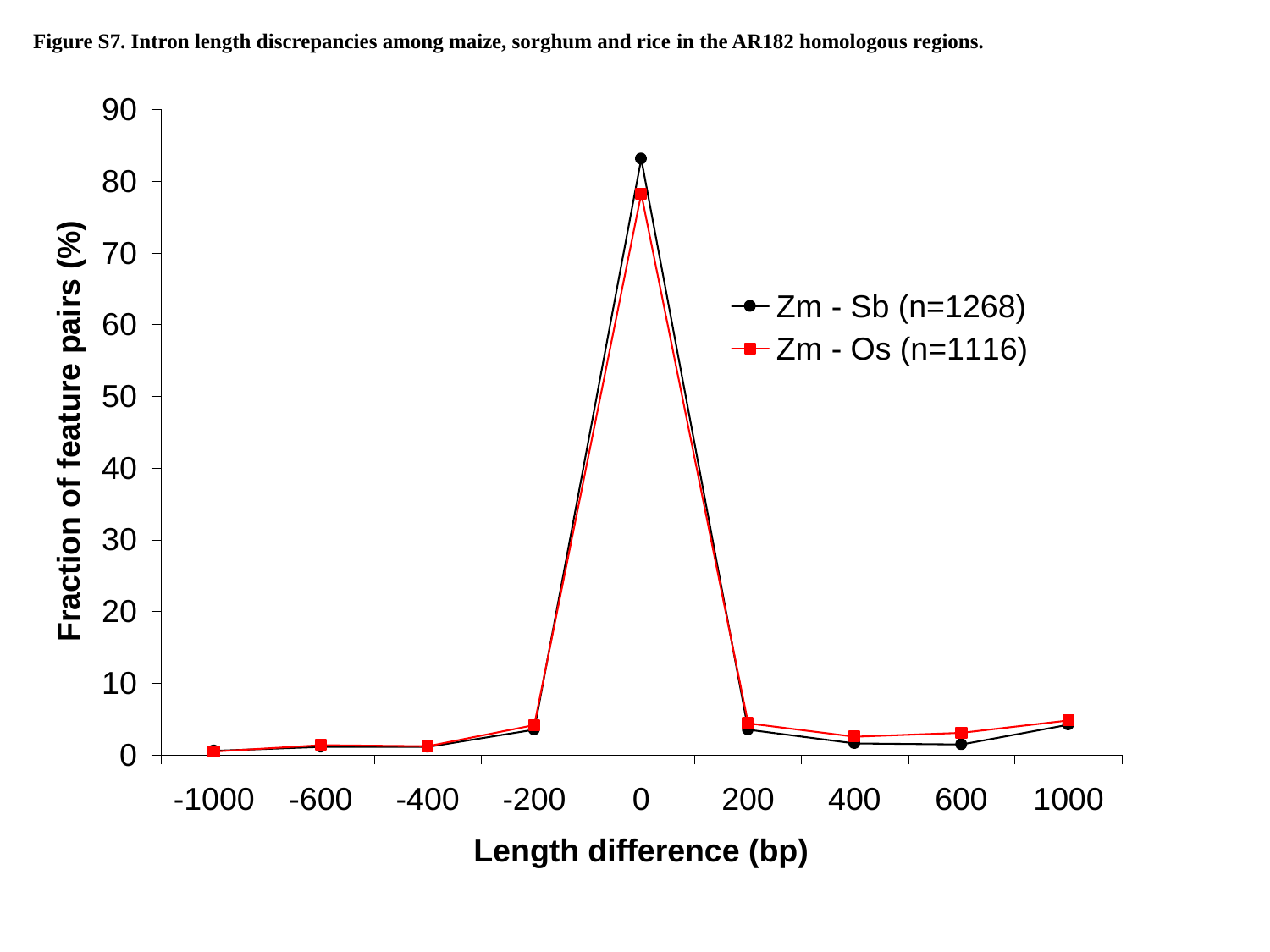

Figure S7. Intron length discrepancies among maize, sorghum and rice in the AR182 homologous regions.
