# Supplementary material for: Detailed Analysis of a Contiguous 22-Mb Region of the Maize Genome
Source: PLoS Genet. 2009 Nov 20;5(11):e1000728. doi: 10.1371/journal.pgen.1000728 (PMC2773423; doi:10.1371/journal.pgen.1000728)
Supplement: Figure S8 — Relationship between sequence length differences in ortholologous introns and the presence of repetitive sequences. Intron length differences are calculated as (length of the maize intron)-(length of the sorghum intron), such that negative values occur when the maize intron is shorter than its ortholog. Each intron length difference is plotted against repetitive content. (0.08 MB PPT) [file pgen.1000728.s008.ppt]

## Slide 1
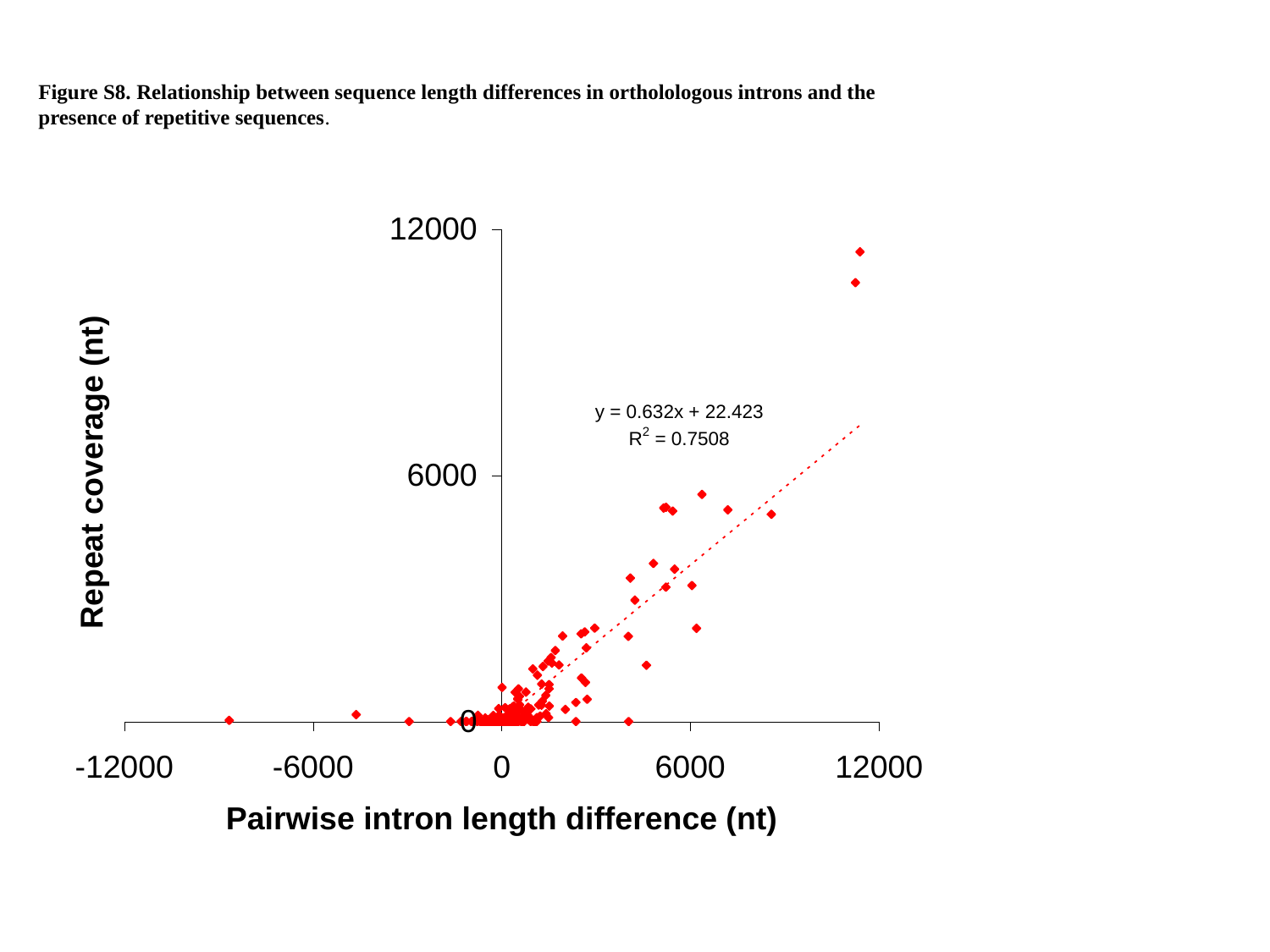

Figure S8. Relationship between sequence length differences in ortholologous introns and the
presence of repetitive sequences.
