# Supplementary material for: Detailed Analysis of a Contiguous 22-Mb Region of the Maize Genome
Source: PLoS Genet. 2009 Nov 20;5(11):e1000728. doi: 10.1371/journal.pgen.1000728 (PMC2773423; doi:10.1371/journal.pgen.1000728)
Supplement: Figure S9 — Example of recent insertions of LTR retrotransposons into the intron of an active maize gene. The depicted gene (ZmAcc7g20001011) encodes a transcript corresponding to the full-length cDNA clone ZM_BFb0042A02 (gb accession BT041740), whose translation product is homologous to members of the haloacid dehalogenase superfamily. Two retrotransposons are shown inserted in a nested fashion into the fourth intron. As determined using K-mer and TEnest software [39],[104], the first was classified as a member of the machiavelli family (Copia superfamily), with a date of insertion estimated at 615 thousand years ago. A second insertion was classified as a member of the jaws family (Gypsy superfamily). Although LTR sequences flank jaws, these were classified as solo LTRs. As shown by TBLASTN alignments, all exons are conserved with corresponding orthologs in sorghum and rice, including those that flank the fourth intron. The exon-intron structure is conserved amongst the three orthologous genes, but whereas the the fourth intron is greater than 12 kb in maize, the corresponding introns are only 647 bp and 264 bp in sorghum and rice respectively. DNA-based alignments (BLASTZ/ChainNet) showed extensive coverage of both exons and introns with syntenic regions of rice and sorghum, but retrotransposon sequences did not align. (0.06 MB PPT) [file pgen.1000728.s009.ppt]

## Slide 1
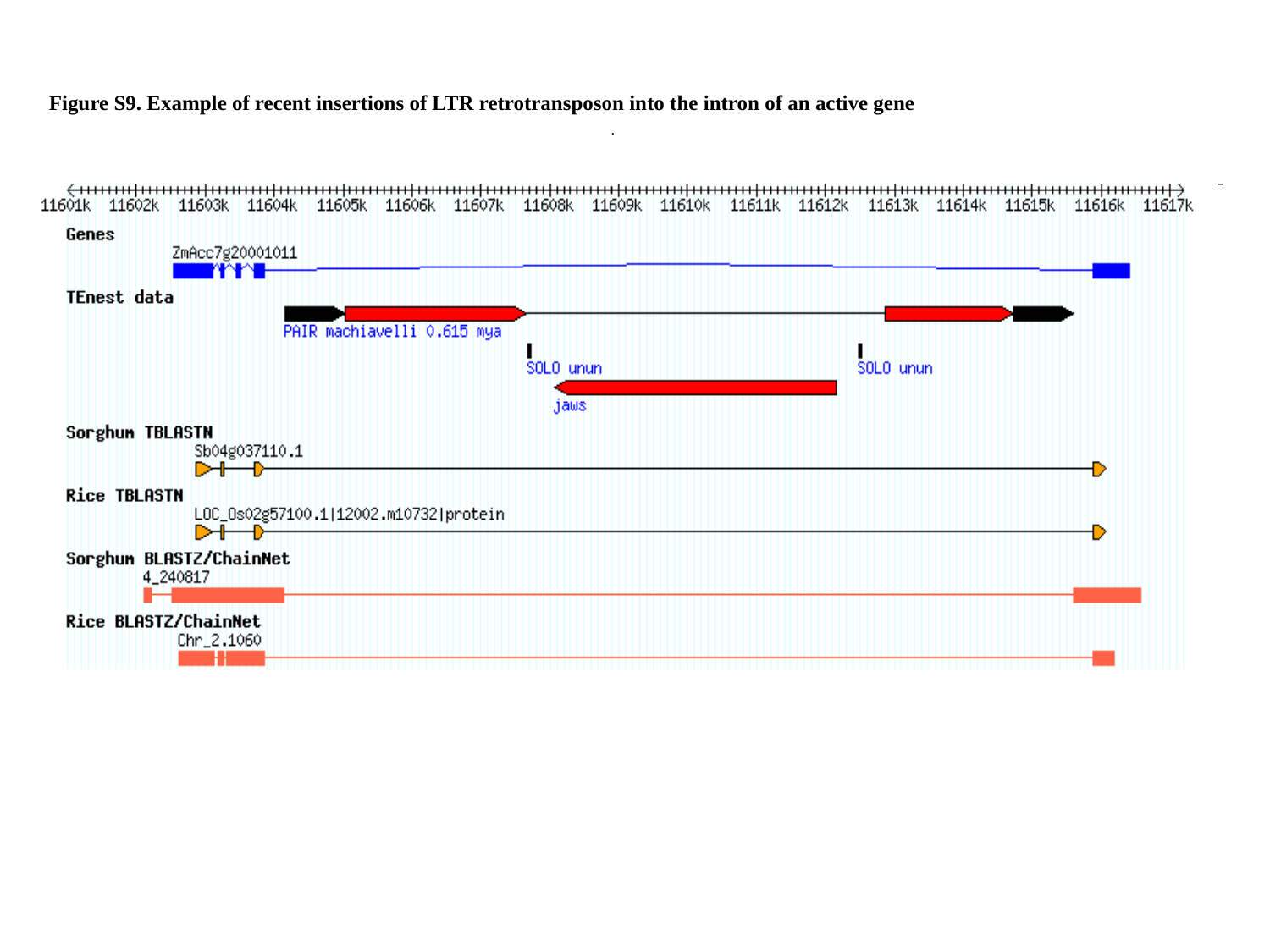

Figure S9. Example of recent insertions of LTR retrotransposon into the intron of an active gene
.
