# Supplementary material for: Detailed Analysis of a Contiguous 22-Mb Region of the Maize Genome
Source: PLoS Genet. 2009 Nov 20;5(11):e1000728. doi: 10.1371/journal.pgen.1000728 (PMC2773423; doi:10.1371/journal.pgen.1000728)
Supplement: Figure S11 — Direct comparison between maize AR182 and its orthologous sorghum pseudomolecules. SyMAP 3 [83] was used to perform the maize pseudomolecule (left) to sorghum pseudomolecule (right) comparisons. (0.34 MB PPT) [file pgen.1000728.s011.ppt]

## Slide 1
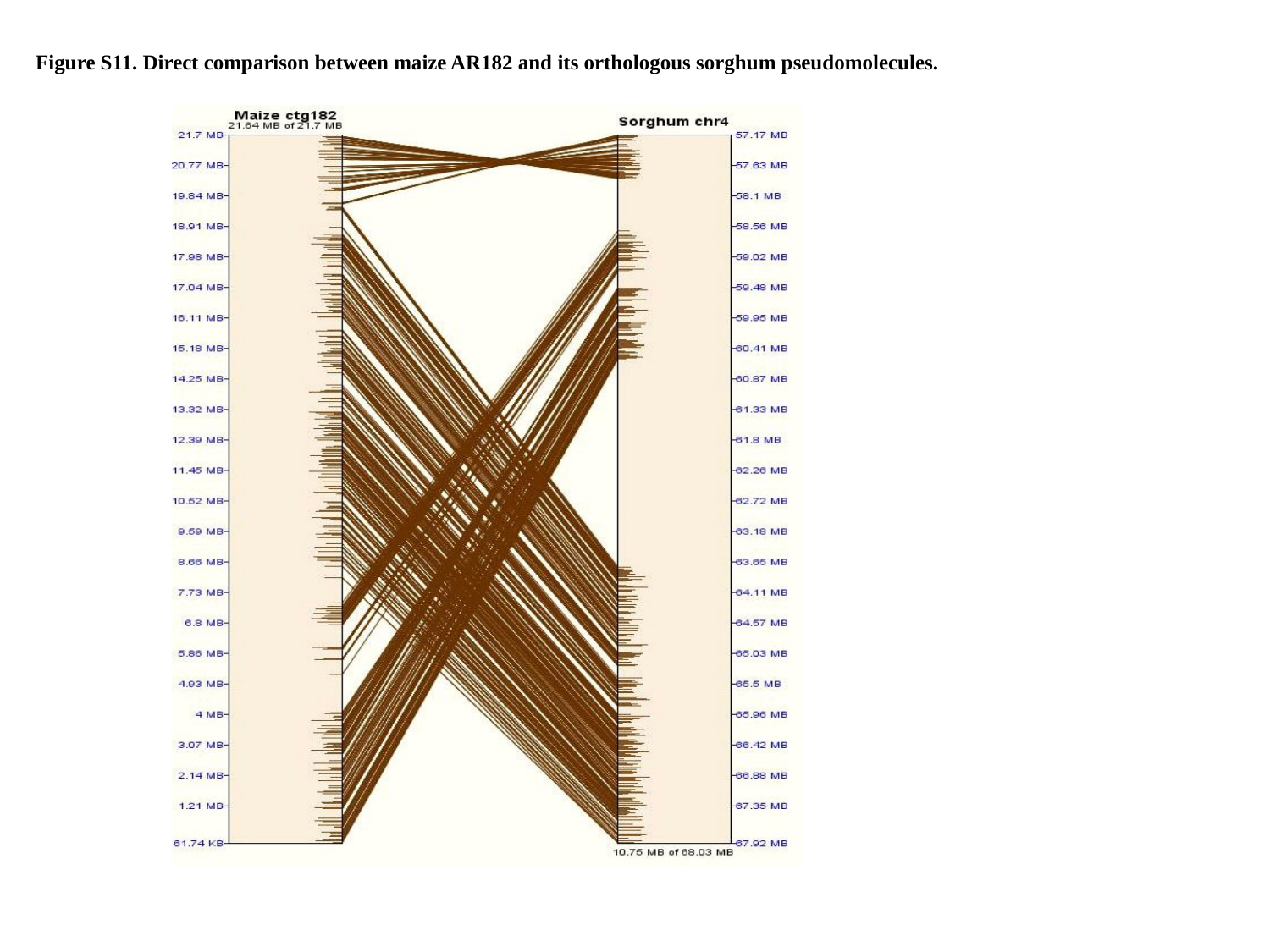

Figure S11. Direct comparison between maize AR182 and its orthologous sorghum pseudomolecules.
