# Supplementary material for: Detailed Analysis of a Contiguous 22-Mb Region of the Maize Genome
Source: PLoS Genet. 2009 Nov 20;5(11):e1000728. doi: 10.1371/journal.pgen.1000728 (PMC2773423; doi:10.1371/journal.pgen.1000728)
Supplement: Figure S14 — Distribution of repetitive elements and small RNAs proximal to gene sequences. (A) Occurrence of repetitive sequences in the upstream of genes (in percentage); (B) Occurrence of repetitive sequences in the downstream of genes (in percentage); (C) Total number of small RNAs in the upstream of genes; (D) Total number of small RNAs in the down stream of genes. (0.14 MB PPT) [file pgen.1000728.s014.ppt]

## Slide 1
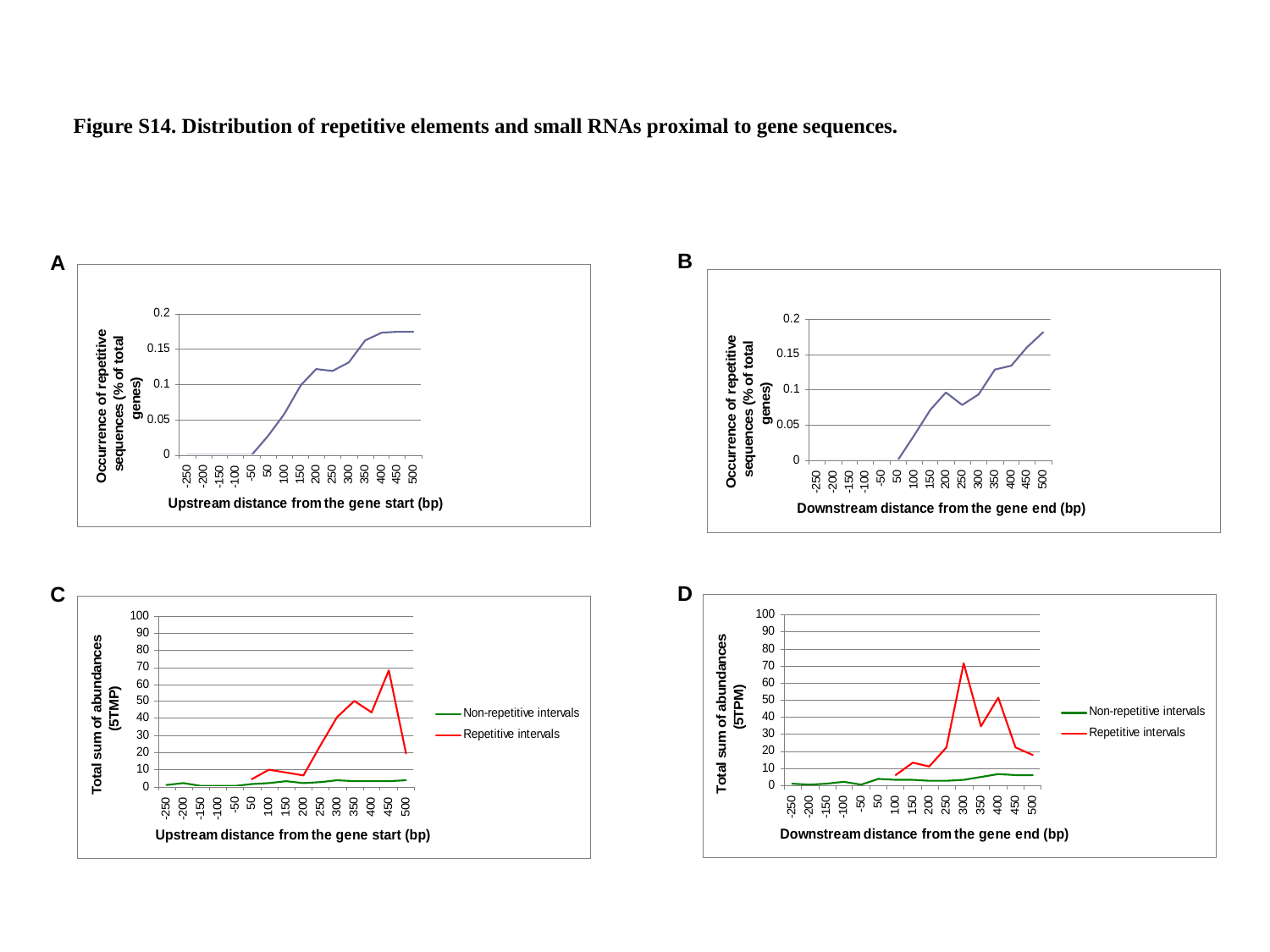

| Figure S14. Distribution of repetitive elements and small RNAs proximal to gene sequences. |
| --- |
B
A
D
C
